# Transcriptomic profile of *TNF^high^* MAIT cells is linked to B cell response following SARS-CoV-2 vaccination

**DOI:** 10.3389/fimmu.2023.1208662

**Published:** 2023-07-26

**Authors:** Paolo Marzano, Simone Balin, Sara Terzoli, Silvia Della Bella, Valentina Cazzetta, Rocco Piazza, Inga Sandrock, Sarina Ravens, Likai Tan, Immo Prinz, Francesca Calcaterra, Clara Di Vito, Assunta Cancellara, Michela Calvi, Anna Carletti, Sara Franzese, Alessandro Frigo, Ahmed Darwish, Antonio Voza, Joanna Mikulak, Domenico Mavilio

**Affiliations:** ^1^Department of Medical Biotechnology and Translational Medicine, University of Milan, Milan, Italy; ^2^Laboratory of Clinical and Experimental Immunology, IRCCS Humanitas Research Hospital, Milan, Italy; ^3^Department of Biomedical Sciences, Humanitas University, Milan, Italy; ^4^Department of Medicine and Surgery, University of Milan-Bicocca, Monza, Italy; ^5^Institute of Immunology, Hannover Medical School (MHH), Hannover, Germany; ^6^Institute of Systems Immunology, Hamburg Center for Translational Immunology (HCTI), University Medical Center Hamburg-Eppendorf, Hamburg, Germany; ^7^Department of Biomedical Unit, IRCCS Humanitas Research Hospital, Milan, Italy

**Keywords:** SARS-CoV-2, mRNA vaccine, MAIT cells, immune response, TNF, B cells, single-cell RNA/TCR-sequencing

## Abstract

**Introduction:**

Higher frequencies of mucosal-associated invariant T (MAIT) cells were associated with an increased adaptive response to mRNA *BNT162b2* SARS-CoV-2 vaccine, however, the mechanistic insights into this relationship are unknown. In the present study, we hypothesized that the TNF response of MAIT cells supports B cell activation following SARS-CoV-2 immunization.

**Methods:**

To investigate the effects of repeated SARS-CoV-2 vaccinations on the peripheral blood mononuclear cells (PBMCs), we performed a longitudinal single cell (sc)RNA-seq and scTCR-seq analysis of SARS-CoV-2 vaccinated healthy adults with two doses of the Pfizer-BioNTech *BNT162b2* mRNA vaccine. Collection of PBMCs was performed 1 day before, 3 and 17 days after prime vaccination, and 3 days and 3 months following vaccine boost. Based on scRNA/TCR-seq data related to regulatory signals induced by the vaccine, we used computational approaches for the functional pathway enrichment analysis (Reactome), dynamics of the effector cell-polarization (RNA Velocity and CellRank), and cell-cell communication (NicheNet).

**Results:**

We identified MAIT cells as an important source of TNF across circulating lymphocytes in response to repeated SARS-CoV-2 *BNT162b2* vaccination. The *TNF^high^* signature of MAIT cells was induced by the second administration of the vaccine. Notably, the increased *TNF* expression was associated with MAIT cell proliferation and efficient anti-SARS-CoV-2 antibody production. Finally, by decoding the ligand-receptor interactions and incorporating intracellular signaling, we predicted *TNF^high^* MAIT cell interplay with different B cell subsets. In specific, predicted *TNF*-mediated activation was selectively directed to conventional switched memory B cells, which are deputed to high-affinity long-term memory.

**Discussion:**

Overall, our results indicate that SARS-CoV-2 *BNT162b2* vaccination influences MAIT cell frequencies and their transcriptional effector profile with the potential to promote B cell activation. This research also provides a blueprint for the promising use of MAIT cells as cellular adjuvants in mRNA-based vaccines.

## 1 Introduction

Mucosal-associated invariant T (MAIT) cells are unconventional T cells defined by their semi-invariant αβ T cell receptor (TCR) composed of TCR α-chain Vα7.2 (*TRAV1-2*) joined to Jα33 (*TRAJ33*) and paired in humans with TCR β-chain V_β_2 (*TRBV20*) and V_β_13 (*TRBV6*) (1–3). The TCR of MAIT cells is restricted to the non-polymorphic major histocompatibility complex (MHC) class I-like protein MR1 that recognizes bacterial- and yeast-derived riboflavin metabolites (4, 5). MAIT cells are abundant in peripheral tissues including the liver and the gut (6, 7), and represent up to 10% of all peripheral blood (PB) circulating T lymphocytes (8). MAIT cells provide a rapid, innate-like effector response to several microbial infections, which leads to migration, proliferative expansion, and cytotoxic activity with the release of effector molecules such as granzymes, perforin, and cytokine secretion such as tumor necrosis factor (TNF), interferon (IFN)-γ, and interleukin (IL)-17 (2, 9–11). In addition, MAIT cells can be activated in an MR1-independent manner via toll-like receptor (TLR) ligands (DNA, RNA, and LPS), IFN-α/β and IL-12, IL-18, and IL-15 (10, 12–14). Cytokine-dependent activation of MAIT cells has been shown in a range of viral infections including Dengue fever (DENV), influenza, hepatitis C virus (HCV), human immunodeficiency virus (HIV), Hantavirus, and, recently, SARS-CoV-2 (15–20).

MAIT cells have been shown to enhance B cell functionality. Indeed, experimental approaches *in vitro* provided evidence that MAIT cells are capable of promoting the differentiation of memory B cells into plasmablasts and favouring antibodies (Ab) production (21, 22). Moreover, both *in vivo* infection (Vibrio cholerae, Simian immunodeficiency virus (SIV)) and vaccination (Shigella dysenteriae, SIV) increase MAIT cell frequencies and their cytokine secretion that positively correlate with B cell activation, specific Ab production, class switching, and memory establishment (23–25). On the other hand, the activation status of MAIT cells correlates with auto-Abs production in different autoimmune diseases. Indeed, an auto-Ab reduction was observed upon MAIT cell inhibition in MR1-deficient mice or upon MR1-inhibitory ligand binding (22, 26, 27).

Recently, higher frequencies of MAIT cells were associated with an increased adaptive response to mRNA *BNT162b2* SARS-CoV-2 vaccine (28). However, the molecular patterns and dynamics of this response are unknown. In the present study, we conducted a comprehensive, longitudinal single-cell RNA-sequencing (scRNA-seq) and scTCR-seq analysis of MAIT cell activation tracked in the PB of healthy adults who received two doses of *BNT162b2* vaccination. We observed that, among PB lymphocytes, *TRAV1-2*^+^ *KLRB1*^+^ MAIT cells represent an important source of TNF after the booster vaccine. This increased expression of *TNF* in MAIT cells correlates with their activation and polyclonal proliferation. Finally, we provide a map of predicted interactions between MAIT and B cells established upon vaccination, indicating a possible TNF-dependent downstream signaling in conventional switched memory B cells.

## 2 Materials and methods

### 2.1 Study design

This study was designed to assess MAIT cell immune responses after immunization with two doses of Pfizer-BioNTech *BNT162b2* mRNA vaccine in individuals without previous SARS-CoV-2 infection. We used scRNA-seq paired with scTCR-seq techniques to uncover the dynamics of MAIT cells upon repeated *BNT162b2* vaccination. In specific, we performed a longitudinal study on six volunteers, collecting peripheral blood mononuclear cell (PBMCs) samples (*n=28*) 1 day before (P0), 3 and 17 days after prime vaccination (P1 and P2, respectively), and 3 and 99 days following vaccine-boost (P3 and P4, respectively).

### 2.2 PBMCs isolation

Freshly PBMCs were isolated through Lympholyte^®^-H Cell Separation density gradient solution (Cedarlane Laboratories, Burlington, North Carolina, USA) according to the manufacturer’s instructions and frozen in Fetal Bovine Serum (FBS, EuroClone) supplemented with 10% of the cryoprotective dimethyl sulfoxide (DMSO, PanReac AppliChem) as previously published (29).

### 2.3 Anti-SARS-CoV-2 IgG Ab titration

Anti-SARS-CoV-2 IgG Ab titration was performed at the Humanitas Research Hospital by a ready-to-use ELISA (enzyme-linked immunosorbent assay) kit (Ref. no. COV19G.CE) for diagnostic use (DIA.PRO; Diagnostic Bioprobes Srl, Italy) and following the manufacturer’s procedures. The IgG Ab levels were measured by the optical density (OD) of 450/620-630 nm. The cutoff OD was evaluated by the formula: cutoff OD= negative control (NC) + 0.250. The specific concentration of IgG was evaluated by the kit-provided standards.

### 2.4 Single-cell RNA and TCR library preparation

Libraries for scRNA-seq were prepared using the Chromium Single Cell Platform with a Single Cell 5′ Library and Gel Bead Kit (10X Genomics, 1000006). Thawed PBMC samples were evaluated for viability prior to scRNA-seq analysis, and all were ≥95% viable. Cells were resuspended in a volume equivalent to 10,000 target cells for each sample and were individually loaded onto a Chromium single-cell controller (10X Genomics) to generate single-cell gel beads-in-emulsion (GEMs). Captured cells were then lysed and the released RNA was barcoded through reverse transcription in individual GEMs. Complementary DNAs (cDNA) were generated and split to generate additional libraries of αβ TCR amplicons. Complementary DNAs were amplified, and the quality was assessed using an Agilent 4200 Tapestation.

The scRNA and scTCR libraries were sequenced using an Illumina Novaseq6000 sequencer with a paired-end 150-bp (PE150) reading strategy (performed by CapitalBio Technology).

### 2.5 Processing scRNA-seq data

The scRNA-seq reads were aligned to the GRCh38 (version refdata-gex-GRCh38-2020-A, 10X Genomics) human genome reference, and UMIs were quantified using Cellranger 5.0.0 (10X Genomics). Subsequent analyses were performed using the Python package Scanpy v1.8.1 (30), under Python v3.8 if not stated otherwise. Raw data matrices of all samples were merged and cells with fewer than 600 expressed genes, or 1,200 UMIs, greater than 8% mitochondrial genes were removed. Data were then log-normalized with a scale factor of 10,000. Highly variable genes (HVGs, 2,000) were identified using the Seurat dispersion-based methods. Data from each sample (*n=28*) were integrated by the Scanpy package. In brief, principal component analysis (PCA) was performed using HVGs via ARPACK implementation of singular value decomposition (SVD). Principal components (PCs) were used to integrate data from different samples by the Harmony algorithm using the Harmonypy package v0.0.6 (31). Neighbors were identified using the top 50 components of Harmony-corrected PCA embeddings, and the clustering analysis was performed using the Leiden algorithm (32) with an initial resolution of 0.1. The clusters were then embedded in two dimensions by using Uniform Manifold Approximation and Projction (UMAP) algorithm. (33).

### 2.6 Cluster marker identification and cell-type annotation

Differential expression gene (DEG) analysis between clusters was carried out to find markers for each of the identified clusters by using the Wilcoxon rank-sum test (Scanpy). Genes with an adjusted p-value < 0.05 and expressed by at least 10% of cells in the cluster at a minimum Log_2_-FoldChange of |0.25| were considered significant. A total of 147,160 CD3^+^ cells were identified based on the expression of canonical cell-type markers and subjected to a next round of dimensionality reduction, and unsupervised clustering as described above. From CD3^+^ T cells, a total of 6,290 MAIT cells embedded in cluster 9 were profiled and annotated based on the expression of canonical cell-type markers, and further re-clusterized. For B cells, a total of 21,013 cells embedded in clusters 7, 11, 33, and 32 were profiled and annotated based on the expression of canonical B cell markers, and further re-clusterized.

### 2.7 Reactome pathway analysis

Pathway enrichment analysis was performed using the *Reactome* web tool (https://reactome.org/) (34). The Reactome pathways of cell type were enriched using DEGs with adjusted p-value < 0.05 and Log_2_-FoldChange > |0.25|. Only enriched terms with FDR < 0.05 were selected as significant and visualized by R package ggplot2 under R v4.2.1. Dots are colored by FDR values and sized by the number of DEGs enriched in each pathway.

### 2.8 RNA velocity analysis

RNA velocity analysis (35) was performed using the Python package scVelo v0.2.4 to infer the future states of a cell from unspliced and spliced mRNA counts. The matrices of spliced and unspliced counts were quantified using the Python library velocyto v0.17 from the aligned bam file generated by CellRanger. Counts matrices in loom file format were loaded to scVelo and merged into the anndata object. Genes detected in less than 20 unspliced and spliced counts were filtered out, and the counts were then normalized. The *filter_genes_dispersion()* function was used to identify the top 2,000 HVGs. The *scv.pp.moments()* function was used to compute the first- and second-order moments among nearest neighbors in the PCA space. The velocities and velocity graphs were estimated using the generalized dynamical model. Velocities were visualized on UMAP embedding. Initial and terminal states were computed by using the Python package CellRank v1.5.0 (36) (*cr.tl.initial_states*, *cr.tl.terminal_states*). The latent time was computed using the scVelo function *scv.tl.recover_latent_time()* by using the CellRank initial and terminal states.

### 2.9 scTCR-seq analysis

The scTCR-seq reads were aligned to the GRCh38 (version refdata-gex-GRCh38-2020-A, 10X Genomics) human genome reference, and consensus αβ TCR annotations were performed using Cellranger VDJ tools 3.1.0 (10X Genomics). Subsequent analyses were performed using the Python package Scirpy v0.10.1 (8) under Python v3.8 if not stated otherwise. TCR annotations were merged with transcriptomics data using the *scirpy.pp.merge_with_ir()* function. TCR quality control was computed by using the *ir.tl.chain_qc()* function; only cells with *TRAV1-2* TCR α chain paired with β chains and cells with TCR α chain *TRAV1-2* paired with β chains plus an additional α and/or β chain were incorporated into the analysis. Paired invariant *TRAV1-2* MAIT cells were detected in 54% of 6,290 MAIT cells. α and β chain combinations were visualized via chord diagrams using the circlize package v0.4.8. Clonotypes were defined according to the amino acid sequence identity of α and β CDR3 regions. Clonal cells were defined as clonotypes that appeared in at least two cells. The alpha diversity of clonotypes was calculated by computing the Normalized Shannon Entropy (Shannon index) (1) within time points. α and β CDR3 sequence lengths were computed by using the *ir.tl.spectratype()* function.

### 2.10 NicheNet analysis

NicheNet analysis was performed using the R package nichenetr v1.1.0 (37) to predict ligand-target links between interacting cells. We applied NicheNet analysis to investigate the cell-cell interactions (CCIs) between MAIT cells, which act as the sender population, and B cells, which act as the receiver population. Both for the sender population and the receiver one, we identified cells at P3. Only genes expressed in at least 10% of the sender or receiver population were considered. The gene sets of interest were identified as the DEGs in B cells between time points P3 and P0 with adjusted p-value < 0.05 and Log_2_-FoldChange > |0.25|. Ligand activities were predicted using the *predict_ligand_activities()* function using the Pearson correlation coefficient and ranked according to the Pearson correlation coefficient. Target genes were predicted by looking at the regulatory potential scores between ligand and target genes of interest, while the ligand-receptor network was predicted by looking at the prior interaction potential. The target genes and the receptor of the top-ranked ligands were visualized via chord diagrams using the circlize package v0.4.8.

### 2.11 DNA proliferation score

The proliferation scores were computed on both total MAIT cells (c9, among all the CD3^+^ lymphocytes) and on subcluster 1 (sc1) of the re-clustering of the MAIT cells. The genes implicated in the DNA replication pathway found in *Reactome* Pathway Analysis (*H3F3B*, *H2AFX*, *DBF4*, *UBB*, *UBE2S*, *UBC*, *FOSB*, and *CCNL1*), with the addition of *MKI67*, were used to calculate the score by the Scanpy-implemented function *scanpy.tl.score_genes().* Null gene expression cells were then excluded from the analysis.

### 2.12 Statistics

Statistical analyses of scRNA-seq data were performed using GraphPad PRISM software version 9.5.0 (La Jolla, California, USA). Statistical differences were calculated with ANOVA tests as specified in the legend. Statistically significant P values were represented with GraphPad style and summarized with the following number of asterisks (*): *P < 0.05; **P < 0.01; ***P < 0.001; and ****P < 0.0001.

### 2.13 Study approval

The enrollable healthy individuals were those who planned to be vaccinated according to the national Italian COVID-19 vaccination program at the Humanitas Research Hospital from November 2020 to April 2021. Recruitment of volunteers was performed according to the Declaration of Helsinki, and all the individuals signed written informed consent. The collection of healthy SARS-CoV-2 vaccinated subjects’ PB samples for research purposes was ethically approved by the Institutional Review Board (IRB) of Humanitas Research Hospital (HRH) (approval 738/20).

## 3 Results

### 3.1 MAIT cells exhibit substantially altered transcriptomes following SARS-CoV-2 vaccination

**Figure 1:**
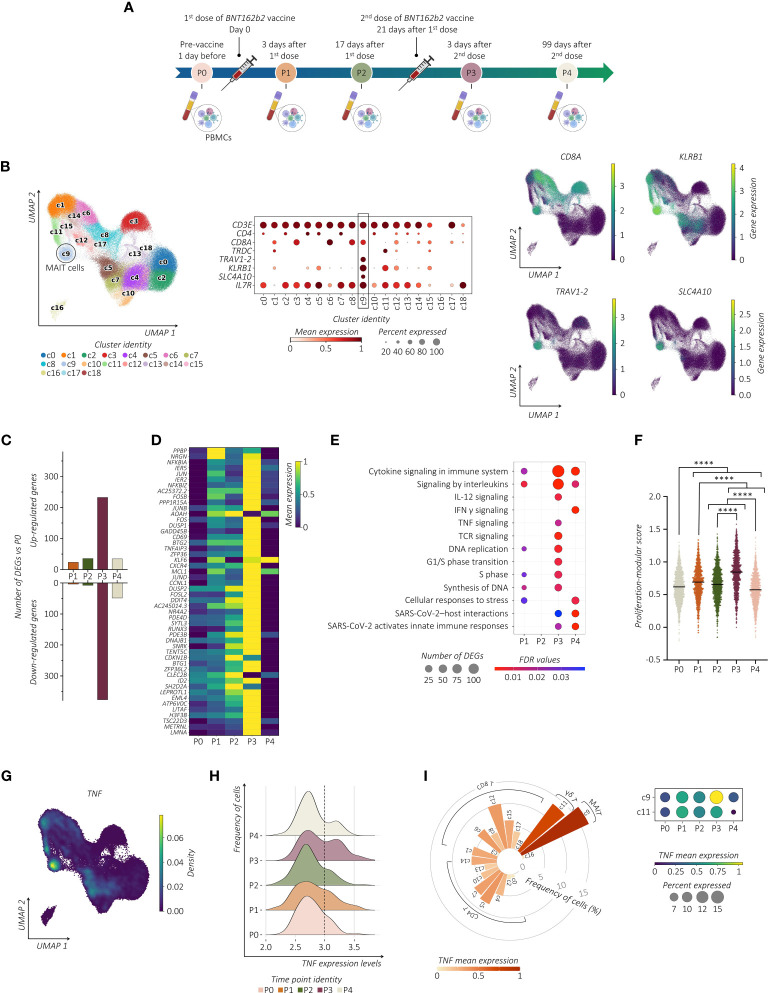
SARS-CoV-2 vaccination shapes the transcriptional pattern of MAIT cells. **(A)** Schematic overview of the experimental design. Collections of PBMCs were performed 1 day before (P0), 3 and 17 days after prime vaccination (P1 and P2, respectively), and 3 and 99 days following vaccine boost (P3 and P4, respectively). **(B)** UMAP clustering (left panel) projection of the integrated PB CD3^+^ T cells from all subjects (s01-s06; a total of 147,160 CD3^+^ cells). The dot plot (middle panel) and the feature plots (right panel) show the expression of canonical markers used for the annotation of MAIT cells. **(C)** The bar plot shows the number of identified DEGs across the different time points compared to P0. For all the figures, DEGs were defined as follows: (i) absolute value of average |log_2_ FC| ≥ 0.25 for upregulated and downregulated genes; (ii) adjusted P values (adj. p) ≤ 0.05, and (iii) detected in at least 10% of cells (min. pct ≥ 10%). **(D)** Transcriptional pattern of MAIT cells at different time points in terms of DEGs. The heatmap shows the gene expression across different time points of the top 50 DEGs (rows) for time point P3 compared to P0. **(E)** The dot plot shows a selection of significantly enriched pathways with FDR values < 0.05, identified among DEGs at each time point for the MAIT cell cluster using the *Reactome* pathway browser. Dots are colored by FDR values and sized by the number of DEGs enriched in each pathway. **(F)** The scatter plot shows the median of the proliferation-modular score of MAIT cells calculated as *AddModuleScore* of nine genes associated with the proliferation signature (*MKI67*, *H3F3B*, *H2AFX*, *DBF4*, *UBB*, *UBE2S*, *UBC*, *FOSB*, and *CCNL1*). The unpaired parametric ANOVA test was used for the statistical analysis, statistically significant *P* value was represented as (*); ****P < 0.0001. **(G)** Kernel density for the *TNF* embedded on UMAP projection. **(H)** The ridge plot shows the *TNF* expression levels (x-axis, log-UMI) and the related cell frequency of cells (y-axis) across the different time points. Null gene expression cells were excluded from the analysis. The dotted line highlights the changes in *TNF* gene expression level across the different time points. **(I)** The circular bar plot (left panel) shows the frequency of *TNF*-expressing cells across all the CD3^+^ clusters, where each bar is colored for the scaled *TNF* mean expression. The dot plot (right panel) shows the expression of *TNF* in both MAIT cell (c9) and γδ T cell clusters (c11) across all the time points.

To investigate the effects of repeated SARS-CoV-2 vaccinations on the transcriptomic profile of the PB circulating MAIT cells, we performed a longitudinal scRNA-seq study on six volunteers vaccinated with two doses of the SARS-CoV-2 Pfizer-BioNTech *BNT162b2* mRNA vaccine. Collection of PBMCs was performed 1 day before (P0), 3 and 17 days after prime vaccination (P1 and P2, respectively), and 3 and 99 days following vaccine boost (P3 and P4, respectively) (**Figure 1A**). Among lymphocytes, MAIT cells were identified as those expressing CD3 (*CD3E*), invariant T cell receptor (TCR) α chain *TRAV1-2* region-encoding segment, and canonical markers CD8 (*CD8A*), CD161 (*KLRB1*), *SLC4A10* (encoding solute carrier family 4 member 10), and CD127 (*IL7R*) (**Figure 1B**; **Supplemental Figure 1A**). In total, we refined 6,290 MAIT cells that UMAP analysis projected to cluster 9 (c9).

Examining the transcriptional pattern of MAIT cells at different time points in terms of differentially expressed genes (DEGs) using P0 as the baseline, we observed a higher number of modulated genes shortly after the vaccine boost (**Figure 1C**). Indeed, MAIT cells at P3 increased expression of early activating marker *CD69* and several members of the transcription factor (TF) families including genes of AP-1 (i.e., *JUN*, *JUNB*, *JUND*, *FOSB*, and *FOSL2*), and NF-kB (i.e., *NFKBIA*, *NFKBIZ*, *NFKB1*, *MAP3K8*, and *RELB*) (**Figure 1D**; **Supplemental Table**). To further provide evidence of MAIT cell responsiveness to vaccines, we used pathway-based analysis instead of the ‘one-gene-at-a-time’ approach by using *Reactome* (https://www.reactome.org) (34). The vaccine boost, compared to the first immunization, provided a much wider response related to the modulation of pathways, including TNF and IFN-γ (encoded by *IFNG*) cytokines, IL-12 signaling, TCR-signaling, and pathways related to SARS-CoV-2 host interactions and activation of the cell cycle when compared to the first immunization (**Figure 1E**). To further provide evidence of cycling MAIT cells, we analyzed the presence of gene proliferation signature score (*MKI67*, *H3F3B*, *H2AFX*, *DBF4*, *UBB*, *UBE2S*, *UBC*, *FOSB*, and *CCNL1*) showing a significant increase after the second dose of vaccine (**Figure 1F**). We then assessed whether the modified cytokine-related pathways were associated with their up- or down-modulation. Longitudinal analysis of the cytokine-specific gene expression showed that, in immunized individuals, MAIT cells were distinguished for high expression levels of *TNF* (**Figure 1G**). Importantly, this *TNF^high^* signature at P3 was a consequence of a progressive increase in *TNF* expression after vaccination (**Figure 1H**). These data implicate MAIT cells as an important source of *TNF* across circulating T lymphocytes after the booster vaccine (**Figure 1I**). Expression of *TNF* in MAIT cells exceeded those of other cells such as gamma delta (γδ) T cells, in which, the high *TNF* expression was unrelated to the second dose of the vaccine, thus indicating that the vaccine-induced activation of MAIT cells differs from other innate-like lymphocytes, although it may not be exclusive. On the contrary, in MAIT cells we found very low expression levels of *IFNG* and IL-12 (*IL12A*) (**Supplemental Figure 1B**). In addition, despite the known propensity of MAIT cells to produce IL-17 (*IL17A*) (11), no expression of this gene was detected.

### 3.2 Polyclonal MAIT cell proliferation following SARS-CoV-2 vaccination converges on the effector TNF polarization

**Figure 2:**
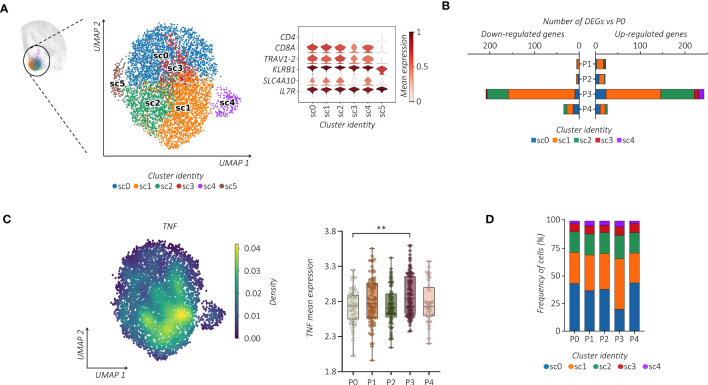
Single-cell tracking of responsive MAIT cells following vaccination. **(A)** UMAP clustering projection (left panel) of the integrated PB MAIT cells from all subjects (s01-s06; a total of 6,290 MAIT cells). The violin plot (right panel) shows the expression of canonical markers used for the annotation of MAIT cells. **(B)** The bar plot shows, for each cluster, the number of identified DEGs across the different time points compared to P0. For all figures, DEGs were defined as follows: **(i)** absolute value of average |log_2_ FC| ≥ 0.25 for upregulated and downregulated genes; **(ii)** adjusted P values (adj. p) ≤ 0.05, and **(iii)** detected in at least 10% of cells (min. pct ≥ 10%). **(C)** Kernel density (left panel) of the *TNF* embedded on UMAP projection. The box plot (right panel) shows the *TNF* mean expression across the different time points in sub-cluster 1 (sc1). The unpaired parametric ANOVA test was used for the statistical analysis, statistically significant *P* value was represented as (*); **P < 0.01. **(D)** The bar plot shows the frequency (%) of cluster distribution across all the time points. Cell numbers were normalized to the total loaded cell number per time point.

Considering the heterogeneity of MAIT cells (38, 39), we performed additional analysis. Notably, all MAIT cells detected in the c9 were subjected to further re-clustering analysis, which generated six sub-clusters (sc0-5) (**Figure 2A**). We centered our analysis on *TRAV1-2*^+^ cells embedded in sc0-4 that differ from each other in their transcriptomic profiles (**Supplemental Figure 2A**). Regarding the kinetics and amplitude of DEGs, we found a heterogeneous response across sub-clusters with major changes detected in sc1 (**Figure 2B**). Indeed, refined *Reactome* pathway enrichment analysis performed for each MAIT sub-cluster confirmed sc1 as the most responsive sub-cluster after the second dose of the vaccine (**Supplemental Figure 2B**). According to our previous observation, cells projected to sc1 showed a higher expression level of *TNF*, which increased after the second dose of the vaccine (**Figure 2C**). Additionally, cells in sc1 showed an enhanced proliferation score over the pre-immunization level, which correlated with an increase in the sc1 relative frequency distribution across MAIT cells (**Figure 2D**; **Supplemental Figure 2C**). Consequently, we found significant DNA-replication-related pathways enriched in sc1 (**Supplemental Figure 2B**).

**Figure 3:**
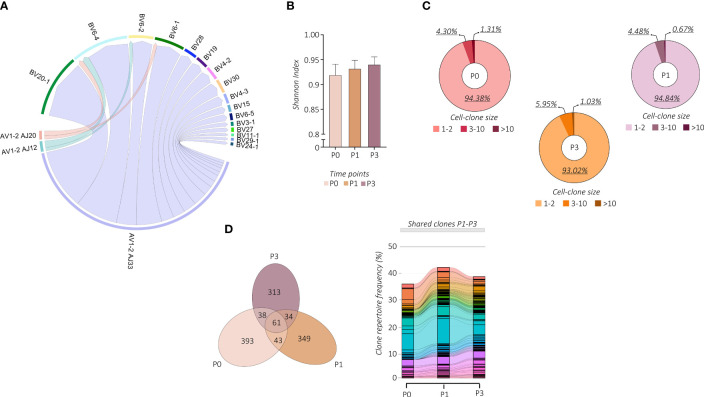
Changes in MAIT cell TCR repertoire following vaccination. **(A)** The chain pairing of different *AVs*/*AJs* pairs with *BVs* chains at all the time points and subjects together are displayed as chord diagrams, where ribbons connecting chains are proportional to the number of paired *AVs*/*AJs* with *BVs* chains. **(B)** MAIT-TCR repertoire diversities were estimated by the normalized Shannon Index ranging from 0 (completely monoclonal) to 1 (completely polyclonal). The bar plot shows the Shannon Index of time points P0, P1, and P3. **(C)** The pie charts show the frequency (%) distribution of the expanded MAIT-TCR clones grouped by cell sizes across time points P0, P1, and P3. **(D)** The Venn diagram (left panel) shows the overlapping MAIT-TCR clonotypes among time points P0, P1, and P3. The alluvion plot (right panel) shows the longitudinal tracking of the overlapping MAIT-TCR clonotypes among time points P0, P1, and P3. Each stratum represents a unique MAIT-TCR clonotype highlighted by a different color. The colored bands between columns represent shared clones among different time points.

We then wondered whether the vaccine-induced proliferation of *TRAV1-2*^+^ MAIT cells modifies their TCR repertoire. We linked our scRNA-seq data to the individual αβ TCR repertoire generated at single-cell resolution. Regardless of vaccination, TCR-encoding segment *TRAV1-2* of analyzed MAIT cells paired with *TRAJ33* and, at much lower frequencies, with *TRAJ12* or *TRAJ20*. In addition, analyzed subjects showed oligoclonal TCRβ chain usage, mainly *TRBV20* and *TRBV6* (**Figure 3A**; **Supplemental Figures 3A, B**). However, the proportion of the TCRβ chain usage was subjected to individual changes upon vaccination (**Supplemental Figure 3C**), indicating the subject-specific alteration of the MAIT TCR repertoire. On the other hand, constant CDR3α and CDR3β sequence lengths were maintained (**Supplemental Figure 3D**). The PB *TRAV1-2*^+^ MAIT cells presented a highly polyclonal TCR repertoire as shown by the Shannon index, ranging from 0 (monoclonal) to 1 (polyclonal), which was maintained after the vaccine boost, indicating a polyclonal proliferation of the MAIT cells (**Figure 3B**). Consequently, we found a prevalence of numerically low cell-size clones (1-3 cells) at all time points (**Figure 3C**). Importantly, this polyclonal proliferation of MAIT cells at P3 was not the consequence of the first vaccination but was rather due to the independent activation upon the boost. Indeed, in tracking the fate of individual MAIT cell clonotypes with regard to shared clones between P0, P1, and P3, we did not find any specific clonotype expansion after the booster vaccine (**Figure 3D**; **Supplemental Figure 3E**).

**Figure 4:**
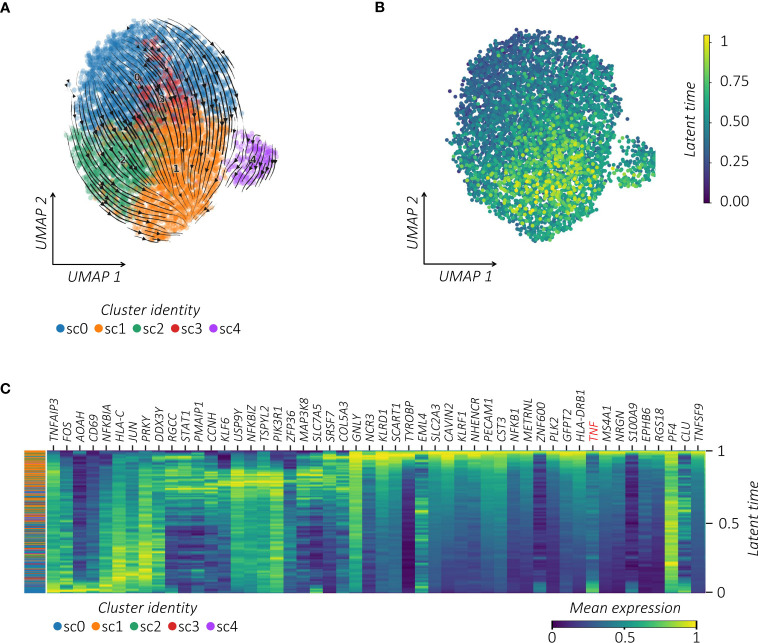
*TNF^high^* MAIT cells converge towards a responsive profile following vaccination. **(A)** RNA velocity projection on UMAP plot. Each dot represents a cell. Cells are colored according to corresponding clusters. The arrows represent the RNA velocity field. **(B)** Latent time projection on UMAP plot. Each dot represents a cell colored according to its latent time value. **(C)** Heatmap of the top 50 latent time-dependent lineage driver genes identified by scVelo. The X-axis represents cells ordered by latent time (from left to right) and different colors correspond to each gene’s scaled (Z-scored) expression in each cell.

To further infer the activation relatedness of MAIT sub-clusters defined above, we estimated the RNA velocity by using the *scVelo* toolkit combined with the *CellRank* algorithm projected on the pre-computed UMAP (35) (**Figure 4A**). This approach, based on the ratio of spliced to unspliced pre-RNA transcripts and transcriptional similarities, is used to identify the directional transitions between cell clusters and hence construct the effector trajectories that account for the speed and direction of motion (35, 40). This analysis visualized the inferred connectivity between MAIT sub-clusters and identified a trajectory with initial and transient states going to the final state, which is characterized by high values of latent time (**Figure 4B**; **Supplemental Figure 4**). From velocity streamlines, we observed that MAIT cells with higher latent time overlapped with highly vaccine-responsive cells in sc1. By correlating gene expression with cell latent time, we visualized gene expression cascades specific to the MAIT cellular trajectory upon vaccination (**Figure 4C**). The top genes driving the pronounced dynamics behavior and ranked from largest to smallest, showed important overlap with the vaccine-related activation profile of MAIT cells (**Figure 1D**) (i.e., *TNFAIP3*, *FOS*, *FOSB*, *FOSL2*, *JUN*, *JUNB*, *CXCR4*, *CD69*, *NFKBIA*, *NFKBIZ*, *KLF6*, and *ZFP36*) to finally converge to their high *TNF* profile.

Taken together, repeated immunizations induced transcriptional changes of MAIT cells linked to their proliferation and to the reinforcement of their effector TNF polarization.

### 3.3 Transcriptional *TNF^high^* state of MAIT cells is linked to B cell response following immunization

**Figure 5:**
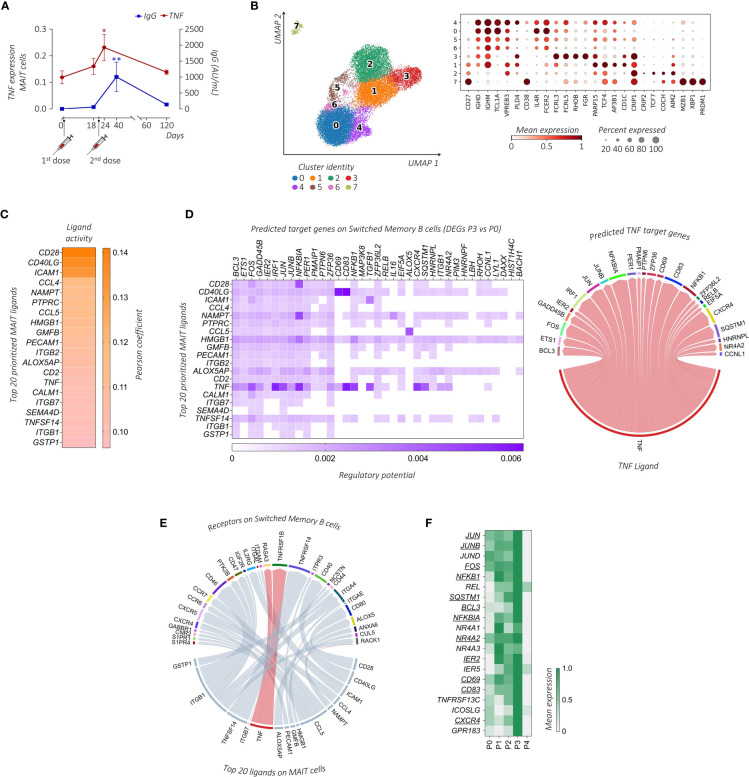
Transcriptional prediction of MAIT and B cells cross-talk following immunization. **(A)** Comparative analysis between the mean *TNF* expression level (red line) and the circulating anti-SARS-CoV-2 IgG concentrations (AU/mL, blue line) of the subject enrolled in the study across the different days post-immunization. The paired parametric ANOVA test was used for the statistical analysis, statistically significant P values were represented as (*): *P < 0.05, **P < 0.01 **(B)** UMAP clustering projection (left panel) of the integrated PB B cells and ASCs from all subjects (s01-s06; a total of 20,722 B cells and 291 ASCs). The dot plot (right panel) shows the expression of markers used for the annotation of the different maturation stages of B cells and ASCs, where clusters have been ordered following the maturation path (*IGHD*, *IGHM*, *TCL1A*, *VPREB3*, *PLD4*, and *CD38* for transitional B cells, enriched in cluster 4; *IGHD*, *IGHM*, *TCL1A*, *IL4R*, and *FCER2* for naïve B cells, enriched in clusters 0, 5, and 6; *FCRL3*, *FCRL5*, *RHOB*, and *FGR* for double negative (DN) B cells, cluster 3; *CD27*, *PARP15*, *TCF4*, *AP3B1*, and *CD1C* for unswitched memory (USM) B cells, enriched in cluster 1; *CD27*, *CRIP1*, *CRIP2*, *TCF7*, *COCH*, and *AIM2* for switched memory (SM) B cells, enriched in cluster 2; *CD27*, *MZB1*, *XBP1*, and *PRDM1* for ASCs, enriched in cluster 7). **(C)** The outcome of NicheNet’s ligand-target pairs regulating genes differentially expressed by the SM B cells between time points P3 and P0 (target genes). The top 20 prioritized ligands expressed by MAIT cells at time point P3, ranking according to the Pearson correlation coefficient, are ordered from higher (top) to lower values (bottom). **(D)** On the left, the ligand-target matrix between the top 20 prioritized MAIT ligands expressed at time point P3 and the predicted target genes expressed by the SM B cells, where the matrix is colored according to the regulatory potential values. On the right, the chord diagram shows the interaction between MAIT-derived *TNF*-ligand at time point P3 with its predicted target genes on SM B cells at P3, where the ribbons connecting chains are proportional to the regulatory potential scores. **(E)** The chord diagram shows the interaction between MAIT-derived ligands at time point P3 with receptors expressed by SM B cells at time point P3, where the ribbons connecting chains are proportional to the prior interaction potential. *TNF*-receptor pairs are highlighted in red. **(F)** The heatmap shows the mean gene expression of maturation, migration, and activation–related genes in the SM B cells across the different time points identified from the NicheNet analysis among the predicted *TNF* target genes (underlined) together with genes found in the literature with similar functions.

Previous findings showed that the frequency of the MAIT cell compartment positively correlates with the anti-SARS-CoV-2 IgG levels in *BNT162b2* mRNA vaccinated subjects (28). Since some SARS-CoV-2 infected or vaccinated individuals may elicit a cellular sensitization without evidence of the virus-specific Ab production (41, 42), we performed a comparative analysis between the concentrations of anti-SARS-CoV-2 IgG and the level of the MAIT-associated *TNF* in our dataset. Importantly, we found an overlapping trend between these two parameters that showed the highest magnitude early after the vaccine boost in relation to their pre-vaccine status (**Figure 5A**).

We then turned to the question of whether MAIT cells can provide help to B cell response. First, we examined B cell populations in our scRNA-seq dataset. Among total PBMCs, B cells and Ab-secreting cells (ASCs) were identified based on the expression of canonical markers including *CD19*, *MS4A1*, *XBP1*, *MZB1*, and *PRDM1* (43). B cells were annotated in clusters 7, 11, and 33, while ASCs were identified in cluster 32 (**Supplemental Figure 1A**). To further characterize these populations, B cells and ASCs were re-clustered and eight (0–7) clusters were obtained (**Figure 5B**). Based on previously published B cell subset transcriptomic signatures (43–46), we annotated cluster 4 as transitional B cells, clusters 0, 5, and 6 as naïve B cells, cluster 1 as unswitched memory (USM) B cells, cluster 3 as double negative (DN) B cells, cluster 2 as switched memory (SM) B cells, and cluster 7 as ASCs.

To investigate the MAIT cell-B cell interaction we used *NicheNet*, an algorithm that infers active ligand-target links between interacting cells (37). Based on the gene expression changes detected upon vaccination at P3, we predicted networks of the ligand-receptor and ligand-target gene links between MAIT cells (defined as the senders) and specific B cell subsets (defined as receivers). Clusters 5-7 of B cells with no significant DEGs upon vaccination were excluded from the *NicheNet* analysis. In order to predict which MAIT cell ligands most likely affected the gene expression in specific B cell subsets, for each couple of sender-receiver cells we ranked the ligand activity, obtaining a list of the top 20 prioritized MAIT ligands (**Figure 5C**; **Supplemental Figure 5**). According to the fundamental role played by CD40L-CD40 in T-B cell interactions, *CD40LG* had a very high activity in the interaction of MAIT cells with all B cell sub-clusters, as assessed by its high-ranking position among the prioritized MAIT ligands. Other MAIT ligands involved in the activation of all B cell subsets included *HMGB1*, *NAMPT*, and *CCL4*, all genes known to be relevant for B cell activation (44, 47, 48).

Focusing on the role of TNF in MAIT-B cell interactions, *TNF* appeared in the list of the top 20 prioritized MAIT ligands only when considering the interactions between MAIT cells and cluster 2, which identifies SM B cells. Importantly, molecular ligand-target gene interactions related specifically to *TNF*-communication were closely linked to the B cell activation program that includes different transcription factors (TFs) (i.e., members of TF families AP-1: *JUNB, JUN*, and *FOS*; NF-κB: *NFKBIA, NFKB1*, and *RELB*; and *IRF1, IER2, ETS1*, and *NR4A2*) and molecules such as *CXCR4*, *CD83*, and *BCL3* (**Figure 5D**). Looking for specific ligand-receptor interaction in SM B cells, we found that *TNF* prioritized the B cell membrane TNF-binding receptor *TNFRSF1B* (TNFR2) (**Figure 5E**), whose expression is a characteristic of memory B cells (49).

Furthermore, we stratified the gene expression of the activation, maturation, and migration molecules identified above, together with known SM B cell-related functional molecules (i.e., activation markers *TNFRSF13C* and *ICOSLG*, and the lymphoid follicles homing marker *GPR183*) (50–52), along the different time points. Notably, the expression of all these markers in the SM B cell cluster was maximally increased at time point P3 (**Figure 5F**), thus suggesting that the *BNT162b2* mRNA vaccine boost can promote MAIT cell activity and favor B cell activation and migration towards lymphoid follicles where the GC reaction occurs.

Taken together, our data reflect a possible TNF-dependent mechanism of MAIT cell action to influence B cell response following mRNA-based vaccination. Moreover, the levels of *TNF* expression in circulating MAIT cells in the PB could serve as a useful, non-invasive predictor cellular biomarker of memory B cell response.

## 4 Discussion

As a component of the innate immune system, MAIT cells respond quickly to infectious agents but have also exhibited activation and increased frequencies in response to vaccination in humans (24, 53), mice (54), and macaques (25, 55). In the present longitudinal study, we evaluated the effects of the mRNA-based vaccine composed of a priming and boosting regimen. At single-cell resolution, we found that the second vaccine-induced polyclonal MAIT cell activation resulted in their increased frequencies and boosted the transcriptional profile of high *TNF* expression.

In this study, many aspects of MAIT cell response were not understood, including the earliest events leading to their activation. Unlike bacteria, the vaccine is unable to generate riboflavin-derived ligands to activate MAIT cells (4, 5), hence we deduced that the ability of MAIT cells to provide immune responses in mRNA vaccine immunogenicity was cytokine-licensed. Indeed, in the boosting phase, several cytokines such as IL-15, IL-12, IL-18, and type-I and II IFNs are released and are important for shaping both humoral and cellular protective immunity (56, 57). Moreover, cytokines, such as IL-15, early after the boost correlated with spike antibody responses (56). Previous observations showed that cytokine-related activation of MAIT cells was associated with increased production of IFN-γ (14, 58). Here, we highlighted the critical role of TNF as an immune effector released by MAIT cells in response to the mRNA-based vaccine, suggesting the specific cytokine pattern of MAIT cell activation in the mRNA SARS-CoV-2 vaccine.

Several new data have shed light on the role of cytokine-driven MAIT cell activation in vaccines. For instance, cytokine-driven activation of MAIT cells was observed in adenoviral-vector platforms (19, 25, 59). The specific mechanism for activation in the adenovirus vaccine has not yet been defined but likely involves lymph nodes as the site of priming (19). This raises another question on the relationship and circulation of MAIT cells between blood and different tissue compartments after mRNA SARS-CoV-2 immunization. The trafficking pattern and relationship of MAIT cells in non-lymphoid tissues, and lymph/lymph nodes, are still under investigation. Some studies provided evidence that MAIT cells located in mucosal barrier tissues are functionally distinct from their blood counterparts in regard to phenotype and functional properties (60, 61). On the other hand, *V. Voillet et al.* provided evidence that MAIT cells are able to egress tissues and enter the lymph during steady-state conditions (62). Consequently, a highly overlapping clonotype usage of MAIT-TCR repertoires was observed in blood and lymph. Therefore, further analysis should be performed to understand whether in our setting the TNF-boosted MAIT cells found in the PB derived from tissue sites.

MAIT cells are capable to support Ab production and provide help to B cells in a vaccine setting. In SIV vaccination, blood and bronchoalveolar lavage (BAL) MAIT cells showed a greater capacity to secrete cytokines/chemokines (i.e., IL-6, IL-8, IL-10, IL-21, TNF, IFN-γ, CCL3, and CCL4) associated with help for B cell activation, migration, and regulation (25). Although the specific role of TNF in this study was not assessed, the culture of MAIT cell derived-supernatants with B cells led to greater tissue-like memory B cell frequencies. TNF is a potent pleiotropic cytokine critical for cell trafficking, inflammation, and host defense against various pathogens. In mammals, TNF has been proven as an important co-stimulator of B cells for their polyclonal expansion on primary immune responses (63). Moreover, TNF is required for the proper development and maintenance of B cell follicles and GC development (64–67).

It is important to notice that TNF inhibitor treatment causes a lower serologic response and impaired memory B cell differentiation due to the repeated mRNA SARS-CoV-2 vaccination. In fact, patients undergoing anti-TNF treatment for chronic inflammatory disease showed a significantly lower humoral response after the SARS-CoV-2 mRNA vaccine (68). After two doses of the mRNA-based *BNT162b2* vaccine, these patients showed defects in the formation of antibody (Ab)-secreting B cells, affinity-matured memory B cells, and a dramatic reduction in Ab longevity (68–73). Therefore, we can hypothesize that TNF plays a crucial role in response to the mRNA-based *BNT162b2* vaccine.

Several recent studies also suggested the adjuvant role of MAIT cells in the context of SARS-CoV-2 vaccination. For instance, a study *in vivo* provided evidence of MAIT cells promoting CD40L-dependent activation of lung-associated dendritic cells (DCs), expansion of follicular T helper (Tfh) cells, and production of antigen-specific mucosal IgA (74). Notably, this mouse model combined with protein antigens from SARS-CoV-2 promoted neutralizing Ab production. Moreover, the flow cytometry approach in *BNT162b2* vaccinated subjects showed that the PB MAIT cell compartment was associated with the magnitude of both the adaptive CD4 T cell response and the humoral response to SARS-CoV-2 spike after vaccination (28). These findings indicated an unexpected association between the PB MAIT cells and the immune response to the *BNT162b2* vaccine. Whereas in the present study, we provided molecular bases that link MAIT and B cell activation upon *BNT162b2* vaccination. In fact, the advantage of having performed a single-cell transcriptional landscape of MAIT cells early (72 hours) after the vaccine boost allowed the detection of a *TNF^high^* activated profile of PB MAIT cells associated with B cell activation and IgG production. Notably, with this approach, we observed that activation of SM B cells, which are the B cell subset responsible for high-affinity long-term memory humoral immune responses, could rely on the effector TNF profile of MAIT cells.

We acknowledge some limitations associated with this study, such as the relatively small number of analyzed individuals, which did not allow us to perform a direct correlation analysis between the TNF response of MAIT cells and the level of anti-SARS-CoV-2 Ab production. In addition, the computational analysis highlighted a possible TNF-mediated mechanism of action of MAIT cells to promote B-cell response that needs to be further tested in an experimental model. Therefore, further studies will be needed not only to confirm the utility of MAIT cells as biomarkers for vaccine response, but also to demonstrate the link between TNF, MAIT, and B cell function in response to the SARS-CoV-2 mRNA-based vaccine.

Overall, our results indicate that SARS-CoV-2 *BNT162b2* vaccination shapes MAIT cell transcriptional effector profile with the potential to promote B cell activation and serve as cellular adjuvants in mRNA-based vaccine platforms.

## Data availability statement

The data presented in the study are deposited in Zenodo, at https://zenodo.org/record/8060366, https://doi.org/10.5281/zenodo.8060366, with accession number 8060366 https://zenodo.org/record/8060366, https://doi.org/10.5281/zenodo.8060366.

## Ethics statement

The studies involving human participants were reviewed and approved by Institutional Review Board (IRB) of Humanitas Research Hospital (HRH) (approval 738/20). The patients/participants provided their written informed consent to participate in this study.

## Author contributions

PM and ST analyzed scRNA/scTCR-seq data, performed statistics, and wrote the manuscript; SB and SDB supervised B cell analysis and wrote the manuscript; RP provided assistance in scRNA/scTCR-seq analysis and in the manuscript’s preparation; IS, SR, LT, and IP designed, performed, and provided assistance in scTCR-seq analysis; VC, AsC, MC, AnC, SF, AF, and AD processed biological specimens and performed experiments; AV, FC, and CV recruited individuals subjected to vaccination, managed sample storage, and assisted in experimental settings; JM and DM equally directed, designed, and supervised the study, analyzed data, and wrote the manuscript. All authors contributed to the article and approved the submitted version.

## Supplementary material

Supplementary Figure 1 Characterization of MAIT cells after the SARS-CoV-2 vaccination. **(A)** Overview of single-cell RNA-seq dataset analysis. UMAP visualization of the integrated single-cell transcriptomes of PBMCs from all the time points from all the subjects (242,765 cells) after QC filtering. In blue are highlighted the CD3^+^ enriched T cell clusters (clusters 1, 3 – 6, 8, 10, 12 – 14, 16 – 19, 23, and 31) identified among all the 36 clusters based on the expression of known markers (CD3 family genes and *CD5*), while in yellow the enriched B cells and ASCs (clusters 7, 11, 33, and 32), identified based on the expression of known markers (*MS4A1* and *CD19* for B cells, and *XBP1*, *MZB1*, and *PRDM1* for ASCs), which have been then re-clusterized. **(B)** The dot plot shows the expression of selected cytokines for each CD3^+^ T cell cluster at time point P3. Dots are colored by the average expression of each gene scaled across all clusters and sized by the percentage of cells within a cluster (min. pct ≥ 10%).

Supplementary Figure 2 Transcriptional profile of the identified MAIT cell clusters. **(A)** The heatmap plot shows the gene expression across different time points of the top 10 DEGs (rows) for each cluster compared to the others. **(B)** The dot plot shows a selection of significantly enriched pathways with FDR values < 0.05, identified among DEGs at each time point compared to P0, for each MAIT cell sub-cluster using the *Reactome* pathway browser. Dots are colored by FDR values and sized by the number of DEGs enriched in each pathway. **(C)** The scatter plot shows the median of the proliferation-modular score in sc1 MAIT cell cluster calculated as AddModuleScore of 9 genes associated with proliferation signature (*MKI67*, *H3F3B*, *H2AFX*, *DBF4*, *UBB*, *UBE2S*, *UBC*, *FOSB*, and *CCNL1*). The unpaired parametric ANOVA test was used for the statistical analysis, statistically significant *P* value was represented as (*); **P* < 0.05; *****P* < 0.0001.

Supplementary Figure 3 Profiling of polyclonal MAIT-TCR repertoire. **(A)** Chain pairing of different *AVs*/*AJs* pairs with *BVs* chains at time point P0 (left panel) and at time point P3 (right panel) for all subjects together are displayed as chord diagrams, where ribbons connecting chains are proportional to the number of paired *AVs*/*AJs* with *BVs* chains. **(B)** The histogram plots show the number of different *AVs*/*AJs* pairs with *BVs* chains across all subjects for all clusters together. **(C)** Chain pairing of different *AVs*/*AJs* pairs with *BVs* chains at time point P0 (left panel) and at time point P3 (right panel) for subject s01, as representative of the cohort, are displayed as chord diagrams, where ribbons connecting chains are proportional to the number of paired *AVs*/*AJs* with *BVs* chains. **(D)** The histograms show the length percentage frequency distribution (%) of CDR3α (left panel) and CDR3β (right panel) across different time points for all clusters together. **(E)** The 20 top-most expanded MAIT-TCR clones for each subject stratified for different time points. Each stratum represents a unique MAIT-TCR clonotype highlighted by a different color. The colored bands between columns represent shared clones among time points.

Supplementary Figure 4 Trajectory inference of MAIT cells. UMAP plot projection of initial and terminal states trajectory identified by CellRank.

Supplementary Figure 5 Cell-cell communication between MAIT cells and B cell populations. The top 20 prioritized ligands expressed by MAIT cells at time point P3 for all the clusters (clusters 5-7 without DEGs were excluded), ranking according to Pearson correlation coefficient, ordered from higher (top) to lower (bottom) values.

Supplementary Table List of differentially expressed genes (DEGs) in MAIT cells at different time points.

## Glossary

**Table:**

Ab	Antibody
adj. p	adjusted P values
AP-1	Activator Protein 1
ASCs	Antibody secreting cells (ASCs)
CDR3	Complementary-determining region 3
COVID-19	SARS-CoV-2 disease 2019
DEGs	Differentially expressed genes
DENV	Dengue fever virus
DN	Double Negative
GC	Germinal center
HCV	Human hepatitis C virus
HDs	Healthy donors
HIV	Human Immunodeficiency virus
HVGs	Highly variable genes
IFN-γ/-α/-β	Interferon gamma/alfa/beta
IL	Interleukin
MAIT	Mucosal-associated invariant T
MHC	Major histocompatibility complex
min.pct	Minimum percentage
MLR	Multiple linear regression
NF-kB	Nuclear factor-kappa B
PB	Peripheral blood
PBMCs	Peripheral blood mononuclear cells
PCA	Principal component analysis
SARS-CoV-2	Severe acute respiratory syndrome coronavirus 2
scRNA-seq	Single-cell RNA
scTCR-seq	Single cell TCR
SIV	Simian immunodeficiency virus
SM	Switched Memory
SVD	Singular value decomposition
TCR	T cell receptor
Tfh	Follicular T helper
TLR	Toll-like receptor
TNF	Tumor necrosis factor
USM	Unswitched Memory
UMAP	Uniform manifold approximation and projection
UMIs	Unique molecular identifiers
αβ T	alpha beta T
γδ T	gamma delta T
