# Supplementary material for: Transcriptomic profile of TNFhigh MAIT cells is linked to B cell response following SARS-CoV-2 vaccination
Source: Front Immunol. 2023 Jul 26;14:1208662. doi: 10.3389/fimmu.2023.1208662 (PMC10410451; doi:10.3389/fimmu.2023.1208662)
Supplement: Supplementary Table — List of differentially expressed genes (DEGs) in MAIT cells at different time points. [file DataSheet_1.pdf]

Significant Differentially Expressed Genes in c9 at each time point vs P0, filtered with pvals_adj values < 0.05 and logfoldchanges (Log2FC) values > 0.25 (up-regulated genes) or < -0.25 (down-regulated genes)

Time point	Genes	logfoldchanges	pvals	pvals_adj
P1	PPBP	1,595809102	8,98661E-13	2,34942E-09
P1	NRGN	1,265467286	1,81495E-05	0,018452507
P1	NFKBIA	0,829109907	4,56517E-40	1,6709E-35
P1	IER5	0,823573768	1,63526E-13	4,60402E-10
P1	JUN	0,785039604	3,51627E-28	4,28997E-24
P1	IER2	0,709131837	1,77033E-21	9,25653E-18
P1	NFKBIZ	0,650509179	7,82125E-08	0,000143133
P1	AC253572.2	0,591575444	1,88031E-06	0,002646969
P1	FOSB	0,577773094	5,2804E-14	1,75698E-10
P1	PPP1R15A	0,551879585	1,43436E-14	5,24991E-11
P1	JUNB	0,524166703	1,11747E-21	6,81673E-18
P1	AOAH	0,520986617	1,5875E-05	0,016601117
P1	FOS	0,48463127	4,59738E-18	2,10336E-14
P1	DUSP1	0,462314516	1,91207E-29	3,49919E-25
P1	GADD45B	0,461795002	3,09967E-06	0,003912106
P1	CD69	0,457141966	2,4313E-22	2,2247E-18
P1	BTG2	0,443057328	1,62207E-07	0,000269861
P1	TNFAIP3	0,405759573	9,47528E-09	1,92669E-05
P1	ZFP36	0,372411489	1,74233E-11	4,25139E-08
P1	KLF6	0,368667006	4,20604E-09	9,05559E-06
P1	CXCR4	0,356793404	1,28589E-13	3,92206E-10
P1	MCL1	0,349723816	4,67128E-07	0,000683894
P1	JUND	0,309234142	1,37472E-05	0,014798846
P1	CCNL1	0,307931721	1,1624E-05	0,012892459
P1	MT-ND3	-0,29516536	1,6053E-10	3,67223E-07
P1	PRF1	-0,298038125	8,77027E-08	0,000152858
P1	SYTL1	-0,418291718	2,81874E-06	0,003684601
P1	TXNIP	-0,450754583	7,72845E-22	5,65738E-18
P2	DUSP2	0,800319552	1,31332E-37	2,40344E-33
P2	NFKBIZ	0,763316512	1,41847E-09	1,85419E-06
P2	CXCR4	0,674014151	2,30624E-41	8,44107E-37
P2	AOAH	0,616912544	1,09551E-07	0,000114562
P2	FOSL2	0,590212882	5,28462E-09	6,66973E-06
P2	DDIT4	0,586350024	1,73361E-10	2,75878E-07
P2	AC245014.3	0,574244499	1,8005E-05	0,012673126
P2	AC253572.2	0,568380594	2,05801E-05	0,013850731
P2	NFKBIA	0,568165302	2,56125E-17	9,37442E-14
P2	NR4A2	0,558968663	1,19651E-08	1,32707E-05
P2	PDE4D	0,546278358	1,99618E-07	0,000192269
P2	SYTL3	0,508110404	2,87238E-06	0,002285476
P2	RUNX3	0,48003763	5,65583E-05	0,031365002
P2	TNFAIP3	0,47452426	1,96E-12	4,21989E-09
P2	PDE3B	0,459589988	3,14058E-05	0,019144236

P2	CD69	0,440246433	4,33743E-18	1,76394E-14
P2	BTG2	0,437302679	2,84458E-07	0,000253938
P2	ZFP36	0,435128033	4,5588E-12	9,26981E-09
P2	DNAJB1	0,408079624	1,82921E-10	2,78962E-07
P2	SNRK	0,398158818	2,59574E-05	0,016380492
P2	TENT5C	0,387116134	3,19062E-05	0,019144236
P2	CDKN1B	0,384136409	4,1266E-07	0,000359613
P2	JUNB	0,369175553	4,12956E-10	6,04584E-07
P2	IER2	0,367187709	1,38399E-06	0,001125679
P2	BTG1	0,358903021	4,52769E-21	2,3674E-17
P2	ZFP36L2	0,343333155	3,05493E-17	1,01649E-13
P2	CLEC2B	0,339353502	2,74434E-05	0,017024691
P2	ID2	0,337231696	4,66197E-06	0,003554849
P2	DUSP1	0,332792014	7,27132E-12	1,40072E-08
P2	SH2D2A	0,319011152	8,46056E-05	0,042419833
P2	LEPROTL1	0,304125428	7,50179E-05	0,038672271
P2	EML4	0,298220962	8,33497E-07	0,000693337
P2	ATP6V0C	0,290409833	6,73281E-05	0,035714134
P2	LITAF	0,276888996	5,06277E-05	0,028953499
P2	H3F3B	0,259019315	1,13185E-12	2,58918E-09
P2	TSC22D3	0,257951677	2,0292E-05	0,013850731
P2	CD52	-0,256105781	1,06585E-08	1,2191E-05
P2	MT-ND3	-0,278951198	3,81459E-11	6,64847E-08
P2	UBL5	-0,281015664	3,4827E-05	0,02055971
P2	PCBP1	-0,315066278	6,31582E-05	0,033994926
P2	ATP5F1E	-0,326025188	3,03401E-13	7,40318E-10
P2	POLR2L	-0,357023686	1,48836E-05	0,010895103
P2	RPS29	-0,362093508	1,34986E-23	1,23515E-19
P2	GIMAP7	-0,400120109	8,31301E-07	0,000693337
P3	METRNL	2,474932432	1,5675E-10	3,33558E-08
P3	LMNA	2,247689724	1,0571E-08	1,79958E-06
P3	NR4A3	2,114849091	1,2659E-15	4,17417E-13
P3	ZNF331	2,108563662	1,53133E-48	1,80801E-45
P3	NR4A2	2,055463791	1,1734E-112	8,5895E-109
P3	MAFF	2,052091837	8,49868E-17	3,27432E-14
P3	CREM	1,969283581	1,19902E-38	1,12527E-35
P3	PER1	1,966150165	1,34858E-52	1,97437E-49
P3	GRASP	1,910583615	1,89703E-05	0,001677129
P3	RBM38	1,865592003	3,52102E-85	1,17157E-81
P3	CD83	1,84689045	1,51177E-09	2,85218E-07
P3	BCL3	1,79202199	3,83373E-19	1,92217E-16
P3	IER3	1,701977491	8,48732E-06	0,000794487
P3	NFKBIZ	1,669927597	2,9383E-48	3,36077E-45
P3	ZC3H12A	1,653798699	2,29813E-19	1,21904E-16
P3	DUSP10	1,643436313	0,000100371	0,007501745
P3	GADD45B	1,632919788	2,24608E-71	4,83581E-68
P3	IER5	1,593770742	3,45763E-52	4,86742E-49
P3	TNFAIP3	1,57596159	1,7367E-140	6,3564E-136

P3	IER5L	1,572775006	0,000103763	0,007719168
P3	NFKBIA	1,547447801	4,1549E-126	3,8018E-122
P3	PFKFB3	1,467568159	4,79126E-14	1,38083E-11
P3	EGR1	1,432008028	0,000110523	0,008172233
P3	H2AFX	1,360825181	6,84102E-11	1,52734E-08
P3	AREG	1,35467267	7,52193E-06	0,000709562
P3	PHLDA1	1,340281487	3,94839E-06	0,000403673
P3	ZFP36	1,319182515	1,138E-100	5,95038E-97
P3	PPP1R15A	1,306923151	3,91075E-78	1,10106E-74
P3	IER2	1,296779752	7,72921E-73	1,88598E-69
P3	BHLHE40	1,286068797	1,50893E-45	1,62436E-42
P3	YPEL5	1,257449389	1,54018E-42	1,5659E-39
P3	CXCR4	1,252328515	3,5789E-139	6,5496E-135
P3	FOSL2	1,245112181	2,77404E-38	2,53831E-35
P3	PDE4D	1,227517009	2,55859E-36	2,22969E-33
P3	NRGN	1,225800991	0,000645996	0,036943924
P3	SLC7A5	1,198504925	1,87368E-17	7,8826E-15
P3	FOS	1,194524288	8,47899E-84	2,58616E-80
P3	PIM3	1,175254583	1,307E-08	2,18437E-06
P3	AL021155.5	1,139998198	0,000247232	0,016422735
P3	DUSP5	1,138867497	5,32397E-12	1,27361E-09
P3	OSER1	1,134504437	6,89114E-06	0,000660269
P3	MAP3K8	1,134181261	6,10511E-24	4,21609E-21
P3	RGCC	1,118084788	1,38954E-10	2,99167E-08
P3	SERTAD1	1,097741365	1,1452E-09	2,21775E-07
P3	PIK3R1	1,095076084	1,10235E-48	1,34491E-45
P3	ARID5A	1,091665268	7,37375E-11	1,63568E-08
P3	PMAIP1	1,083793283	1,31801E-18	6,10641E-16
P3	RELB	1,075843692	5,87834E-09	1,01968E-06
P3	JUN	1,070343375	1,55296E-46	1,72243E-43
P3	WHRN	1,054838419	0,000911852	0,048580334
P3	JOSD1	1,037930846	1,65883E-06	0,000182876
P3	PDE4B	1,025003314	4,21813E-19	2,0585E-16
P3	PPBP	1,024582148	0,000657831	0,037445228
P3	LITAF	1,016592622	2,32444E-58	3,86713E-55
P3	MYADM	1,007424712	1,17883E-18	5,53158E-16
P3	AC020916.1	1,006071091	2,43724E-17	1,0137E-14
P3	PRXL2C	1,006053329	0,000264387	0,017404354
P3	FTH1	1,00574255	1,0736E-138	1,3099E-134
P3	SELENOK	0,998218238	6,77573E-39	6,52628E-36
P3	UBE2S	0,997111142	1,68641E-19	9,21256E-17
P3	MTFP1	0,995826662	1,83171E-16	6,84105E-14
P3	PTGER4	0,99533695	7,65277E-19	3,63765E-16
P3	NFKB1	0,991240442	1,08904E-17	4,74525E-15
P3	CSRNP1	0,98875314	1,48761E-27	1,15847E-24
P3	TUBB4B	0,986926138	5,38652E-16	1,85254E-13
P3	HEXIM1	0,956754625	0,000678123	0,038302462
P3	RUNX3	0,937605202	1,44136E-15	4,66861E-13

P3	SOCS3	0,936216176	8,53716E-14	2,4036E-11
P3	SRGN	0,931076348	3,37623E-89	1,23573E-85
P3	RNF125	0,915093482	2,54259E-19	1,32945E-16
P3	RELL1	0,911960721	4,29622E-05	0,003509954
P3	ELL2	0,911267579	5,95699E-05	0,004638972
P3	CD69	0,909792066	3,33358E-74	8,71516E-71
P3	IRS2	0,909380794	0,000452943	0,027447298
P3	TSC22D3	0,903365374	2,20253E-61	4,03074E-58
P3	GTF3C1	0,902042866	3,83113E-16	1,37474E-13
P3	BTG3	0,900429249	0,000134455	0,009630479
P3	DUSP1	0,891181111	2,76499E-91	1,12446E-87
P3	REL	0,883571088	1,40809E-17	5,99273E-15
P3	GABARAPL1	0,883282602	1,4239E-06	0,000159583
P3	JUNB	0,878557861	8,55246E-54	1,30429E-50
P3	SKIL	0,87373358	6,53572E-06	0,00062951
P3	DUSP2	0,873244643	2,42931E-49	3,06605E-46
P3	SRSF7	0,872479796	7,15424E-54	1,13849E-50
P3	NFE2L2	0,865259111	4,05966E-13	1,06898E-10
P3	TSPYL2	0,862969518	4,04449E-14	1,17486E-11
P3	DNAJB1	0,861386478	1,83182E-42	1,81207E-39
P3	SLC2A3	0,848940849	3,01347E-27	2,29784E-24
P3	SNX9	0,846987784	4,72448E-06	0,000474209
P3	SYAP1	0,844506681	1,63648E-06	0,000180958
P3	IRF1	0,838007092	9,61704E-27	7,18354E-24
P3	MAPK1IP1L	0,83230859	1,15742E-21	7,5648E-19
P3	ETF1	0,831997097	1,12371E-06	0,000129374
P3	TENT5C	0,826494753	4,76844E-20	2,68507E-17
P3	PPP1R15B	0,818623841	3,74611E-05	0,003144758
P3	VPS37B	0,816580236	1,64453E-15	5,27996E-13
P3	DBF4	0,813851058	0,000174833	0,012119432
P3	JMJD6	0,813744009	0,000659696	0,037493088
P3	CITED2	0,796705365	6,07399E-15	1,86819E-12
P3	RGS2	0,793192148	1,41702E-08	2,32575E-06
P3	SYTL3	0,792841136	2,01973E-13	5,47586E-11
P3	DYNLT1	0,786272883	1,19363E-07	1,66114E-05
P3	SAT1	0,785567224	4,96263E-16	1,72988E-13
P3	SBDS	0,77493006	2,56326E-16	9,28892E-14
P3	AC253572.2	0,768845677	2,12938E-10	4,42826E-08
P3	RNF138	0,754548848	1,47732E-05	0,001328533
P3	KLF3	0,750723839	3,91706E-19	1,93741E-16
P3	ARL4A	0,749980271	1,71582E-08	2,79114E-06
P3	JUND	0,744575977	4,36562E-32	3,6315E-29
P3	ZFP36L2	0,731866658	8,23027E-72	1,88273E-68
P3	FOSB	0,725645185	1,77292E-20	1,03001E-17
P3	IFRD1	0,722049475	1,24049E-07	1,71982E-05
P3	IDI1	0,700869799	5,24345E-07	6,61778E-05
P3	SPTY2D1	0,692819297	9,95471E-06	0,000917764
P3	HSH2D	0,68743515	0,000133615	0,009607928

P3	CCNH	0,685354412	8,36894E-16	2,8102E-13
P3	RHOH	0,683072984	4,49593E-11	1,02208E-08
P3	SNHG1	0,67666465	5,67561E-08	8,37633E-06
P3	BTG2	0,674494803	1,32549E-16	5,05356E-14
P3	AC245014.3	0,673445344	3,48752E-08	5,25295E-06
P3	FAM177A1	0,670769751	5,60234E-07	6,97454E-05
P3	HOOK2	0,669494569	2,37711E-07	3,1638E-05
P3	CHD1	0,66082263	4,91153E-12	1,18268E-09
P3	ITPRIP	0,659071982	0,000561589	0,032940266
P3	DNAJB6	0,654830456	7,45598E-10	1,48313E-07
P3	ATG2A	0,651649415	5,38142E-05	0,004254115
P3	GTF2B	0,646804035	3,42468E-05	0,002894839
P3	SNHG3	0,644524455	0,000121553	0,00888016
P3	ZNF267	0,643520892	0,000568175	0,033273232
P3	ZNF394	0,642109096	4,12056E-07	5,25493E-05
P3	BTG1	0,638344586	2,4344E-67	4,95008E-64
P3	DDIT4	0,634852529	2,76264E-13	7,43495E-11
P3	NXT1	0,632783055	0,000255403	0,016873679
P3	CCNL1	0,631759167	2,01395E-21	1,24936E-18
P3	LINC01578	0,620890856	8,71509E-22	5,79965E-19
P3	DCTN4	0,614749074	0,000714259	0,039912335
P3	HSPA5	0,609873593	1,91891E-19	1,03286E-16
P3	TLE1	0,603244126	0,000287297	0,018611281
P3	WHAMM	0,603213251	0,000152719	0,01079089
P3	SRSF2	0,59561336	4,28532E-16	1,50814E-13
P3	CYCS	0,594985485	2,32096E-14	6,79597E-12
P3	JMY	0,589749813	0,000504519	0,030074781
P3	SNHG15	0,583084822	0,000764934	0,041849554
P3	GPR183	0,579966724	0,000704491	0,039608395
P3	ARL4C	0,578592062	5,86798E-50	7,95458E-47
P3	H3F3B	0,576644123	9,10474E-62	1,75391E-58
P3	EML4	0,574322581	1,26312E-21	8,11075E-19
P3	ZBTB1	0,573903203	6,32664E-07	7,74453E-05
P3	MGAT4A	0,573192	2,0125E-16	7,44034E-14
P3	SQSTM1	0,572090268	1,43551E-13	3,98039E-11
P3	PIK3IP1	0,571097076	6,81609E-08	9,74514E-06
P3	EIF1	0,562462807	2,0823E-112	1,2702E-108
P3	RSRC2	0,560704529	1,70609E-06	0,00018696
P3	DNAJA1	0,560701489	1,10881E-07	1,55493E-05
P3	EPB41L4A-AS1	0,553774893	0,000763507	0,041833993
P3	MYLIP	0,542481184	7,18932E-07	8,59923E-05
P3	ID2	0,540282786	6,99524E-15	2,13361E-12
P3	SPOCK2	0,534500062	7,46325E-26	5,46325E-23
P3	KDM2A	0,529917002	3,12562E-05	0,00267918
P3	WTAP	0,52453959	3,07898E-08	4,77515E-06
P3	TIPARP	0,523936033	0,000193792	0,013233179
P3	IRF2BP2	0,517443776	0,000742854	0,040947565
P3	WDR74	0,515974581	0,000406189	0,025070707

P3	POMP	0,515345752	3,94458E-06	0,000403673
P3	HERPUD1	0,513350368	3,25883E-05	0,002773866
P3	TOB1	0,509060383	3,35441E-06	0,000349786
P3	G3BP2	0,507786989	9,15349E-12	2,16146E-09
P3	BCAS2	0,499866247	0,000131171	0,009469375
P3	PDE3B	0,499072254	5,27953E-06	0,000520852
P3	SNHG16	0,496916831	6,81072E-07	8,25428E-05
P3	ATP6V0C	0,495234817	1,23446E-12	3,11604E-10
P3	IFNGR1	0,494198918	3,67799E-06	0,000379206
P3	RNF166	0,490481019	1,21792E-05	0,001111649
P3	CD5	0,490472645	0,00011226	0,00825069
P3	CCDC59	0,489999533	0,000378253	0,023505018
P3	CDK17	0,488949984	1,33286E-08	2,21746E-06
P3	STK17B	0,484590322	5,05691E-14	1,446E-11
P3	RAB8B	0,476960212	0,000298447	0,019163953
P3	ADGRE5	0,471997142	1,60055E-08	2,61525E-06
P3	SNRK	0,464209735	2,70806E-06	0,000287298
P3	LAPTM4A	0,456512153	0,000893895	0,047693072
P3	ZFAND5	0,4553397	0,000424294	0,025925875
P3	PRNP	0,45352599	2,42997E-09	4,44696E-07
P3	AL135905.2	0,453201294	0,00031503	0,020122856
P3	H1FX	0,452473789	2,53238E-05	0,00220685
P3	EIF4A1	0,448637873	4,3726E-19	2,10581E-16
P3	TUBA1A	0,438899517	2,15189E-06	0,000230972
P3	MAP1LC3B	0,431254297	2,52786E-07	3,35225E-05
P3	SURF4	0,429221183	0,000215496	0,014552334
P3	HNRNPA0	0,429126918	7,18829E-12	1,70843E-09
P3	MCL1	0,427817523	3,82126E-10	7,85742E-08
P3	RPL22L1	0,426695973	6,48004E-07	7,90587E-05
P3	LEPROTL1	0,426255345	3,57381E-09	6,4755E-07
P3	SNHG5	0,424975395	6,15378E-08	8,93787E-06
P3	GSPT1	0,420918524	3,91479E-06	0,000402487
P3	DDX6	0,419820875	6,62208E-08	9,5049E-06
P3	DDX24	0,417109221	1,34677E-08	2,23046E-06
P3	RBM8A	0,412115723	1,91699E-07	2,57955E-05
P3	KLF6	0,411081135	1,09601E-10	2,37368E-08
P3	P2RY8	0,408134729	2,668E-08	4,20912E-06
P3	ODC1	0,405255705	0,000580886	0,033801305
P3	DDX3X	0,403109252	3,61299E-07	4,65631E-05
P3	CD44	0,390563041	2,12226E-10	4,42826E-08
P3	UBC	0,37828666	5,31221E-35	4,52168E-32
P3	EIF5A	0,366562545	2,3086E-09	4,24608E-07
P3	RORA	0,364011556	6,37918E-08	9,1923E-06
P3	B4GALT1	0,360931098	0,000419143	0,025740031
P3	HNRNPUL1	0,354711533	6,21712E-07	7,65864E-05
P3	BZW1	0,352150798	9,62681E-08	1,36043E-05
P3	UBALD2	0,346248269	0,000876987	0,047134497
P3	ATP6V1G1	0,334874541	2,89536E-08	4,53098E-06

P3	SFPQ	0,326497555	1,4451E-06	0,000160767
P3	UBB	0,318122953	6,84363E-11	1,52734E-08
P3	KMT2E	0,316782117	6,95917E-06	0,000665046
P3	RBM39	0,311557144	1,11188E-08	1,88407E-06
P3	SRSF3	0,304806858	1,06672E-06	0,000124341
P3	IDS	0,302491844	5,3513E-05	0,004239458
P3	LDHA	0,30045405	5,22945E-06	0,000517305
P3	HNRNPL	0,30034101	3,91738E-05	0,003258634
P3	SNU13	0,299558699	0,00060099	0,034860255
P3	RBM3	0,295731694	5,6764E-09	9,98855E-07
P3	KLF2	0,286168933	1,96218E-09	3,66417E-07
P3	EIF5	0,283334345	2,87495E-05	0,00248761
P3	SARAF	0,281412452	1,74548E-14	5,19402E-12
P3	TAGLN2	0,281034172	4,45001E-06	0,000449931
P3	EPC1	0,280105293	0,000347833	0,021799698
P3	BCLAF1	0,280098259	1,77122E-05	0,001569692
P3	SLC25A5	0,275844127	6,29925E-05	0,004884722
P3	BRD2	0,264393508	0,000294317	0,018931969
P3	EZR	0,256474674	4,19552E-05	0,003435355
P3	RSRP1	0,250855416	0,000574285	0,033470394
P3	SLC38A1	0,250822276	0,000331815	0,020903227
P3	HNRNPA1	0,250094593	1,43602E-15	4,66861E-13
P3	GZMK	-0,250447661	8,3912E-08	1,19041E-05
P3	ARPC2	-0,255577356	2,79269E-06	0,00029542
P3	RAP1B	-0,256947666	0,000376692	0,02344777
P3	TMBIM6	-0,263330251	0,000157683	0,011077443
P3	UQCR11	-0,270457685	0,000205556	0,013932531
P3	OAZ1	-0,27157864	6,23555E-07	7,65864E-05
P3	CD52	-0,271953344	1,17652E-09	2,26641E-07
P3	RPS29	-0,272672474	2,45578E-16	8,98839E-14
P3	EVL	-0,282980919	1,55591E-05	0,001395777
P3	PTPRC	-0,287062228	1,25795E-12	3,15358E-10
P3	TMEM258	-0,289211661	0,000503281	0,030049925
P3	PSMB8	-0,290510327	0,000754289	0,041390883
P3	ITGB2	-0,297865301	1,18912E-06	0,000136284
P3	CNBP	-0,299766302	2,75374E-07	3,62553E-05
P3	CELF2	-0,300322086	0,00035646	0,022302229
P3	CYFIP2	-0,30101788	0,000547234	0,032201455
P3	PRF1	-0,303700954	4,8435E-06	0,000483044
P3	CD3D	-0,303730756	7,80327E-08	1,11131E-05
P3	COMMD6	-0,305025935	2,22291E-05	0,001955786
P3	PSME2	-0,309523195	7,56985E-05	0,005748218
P3	ARPC1B	-0,311097771	4,78625E-06	0,000478638
P3	CLIC1	-0,311727524	1,35719E-06	0,000152844
P3	PTPRCAP	-0,31380859	1,03444E-17	4,56162E-15
P3	ZAP70	-0,314608514	2,24027E-05	0,001966335
P3	OSTF1	-0,315058231	0,000403731	0,024961089
P3	GZMA	-0,317131847	7,03305E-10	1,40665E-07

P3	BRK1	-0,320157111	0,000111487	0,008210301
P3	ATP5PB	-0,320308685	0,000881503	0,047307736
P3	CDC42SE1	-0,33126536	0,000388977	0,02408957
P3	SPTAN1	-0,333021045	0,000530956	0,031344378
P3	MT-ND3	-0,334006369	3,40884E-13	9,04108E-11
P3	SPCS2	-0,334819734	0,000172906	0,012008575
P3	THRAP3	-0,341283262	0,00086793	0,04671634
P3	CLEC2B	-0,343132228	0,000254891	0,016870257
P3	ATP5MF	-0,345004529	0,000724228	0,040407707
P3	MYL12A	-0,34511447	5,41576E-16	1,85254E-13
P3	FYB1	-0,350161523	5,03462E-05	0,004014643
P3	RAC2	-0,350351036	1,99835E-11	4,6587E-09
P3	DAD1	-0,351945847	3,81706E-05	0,003189689
P3	NONO	-0,352985114	0,000245935	0,01636631
P3	SUN2	-0,353915542	6,24E-08	9,02729E-06
P3	IL10RA	-0,354401052	0,00021152	0,014310222
P3	CAPZA1	-0,35548532	0,000733104	0,040655066
P3	TRAPPC1	-0,35691601	0,000363855	0,022687346
P3	SHISA5	-0,358533204	0,000328985	0,02076065
P3	SYTL2	-0,359700292	0,000170822	0,011886443
P3	OCIAD1	-0,366475105	0,000736698	0,040790321
P3	APMAP	-0,366983056	0,000290144	0,018729384
P3	REEP5	-0,368474066	0,000467082	0,028071687
P3	SSU72	-0,36882472	0,000518934	0,030733826
P3	NDUFB10	-0,370187253	0,000339215	0,021332638
P3	ATM	-0,370861173	2,57053E-05	0,002234774
P3	FCMR	-0,372891575	5,89751E-06	0,000575613
P3	PTGES3	-0,37336424	7,3721E-06	0,000699032
P3	TC2N	-0,374012589	4,09525E-07	5,24092E-05
P3	STK4	-0,375667512	2,66556E-06	0,000283611
P3	ATP5MD	-0,377540797	0,00010576	0,007835883
P3	TMC6	-0,379556715	6,27035E-05	0,004872633
P3	PPP1CA	-0,380981565	5,31262E-07	6,68203E-05
P3	ANXA2	-0,382090986	0,000150258	0,010637546
P3	EVI2B	-0,382123411	0,000288126	0,018631998
P3	SLC4A10	-0,382600278	7,14014E-05	0,005433185
P3	CD164	-0,383271575	3,25883E-05	0,002773866
P3	PSMB10	-0,384146333	4,10214E-05	0,003381589
P3	KRTCAP2	-0,386016995	1,6259E-07	2,21225E-05
P3	KMT2A	-0,387094557	0,000146459	0,010429112
P3	CCT6A	-0,387595356	0,000529714	0,03132158
P3	EMP3	-0,388219833	1,0811E-09	2,116E-07
P3	BTN3A2	-0,390921593	0,000342916	0,02152839
P3	HADHA	-0,391726136	0,000323001	0,020431694
P3	HIST1H4C	-0,394111186	0,000146832	0,010435328
P3	SELENOT	-0,395789117	4,33938E-05	0,00353731
P3	CDC25B	-0,398572236	0,000238609	0,015936752
P3	PSMB9	-0,399930924	1,79802E-07	2,42839E-05

P3	IKZF1	-0,400100976	3,91225E-07	5,02429E-05
P3	UQCR10	-0,401720047	6,77066E-06	0,000650428
P3	COX7A2L	-0,403836519	8,72504E-05	0,00659227
P3	SKAP1	-0,404390395	4,22499E-06	0,000428362
P3	GPX4	-0,405768514	2,20728E-06	0,000235535
P3	PTPN4	-0,406471014	0,000230865	0,01547602
P3	TMC8	-0,406891704	7,03532E-05	0,005375774
P3	ABRACL	-0,408372015	7,13445E-06	0,000680021
P3	ATP5F1E	-0,408876568	5,53689E-16	1,87644E-13
P3	CCT8	-0,409320801	0,000732494	0,040655066
P3	DHRS7	-0,410133451	7,33854E-06	0,000697656
P3	SNRNP200	-0,410156578	0,000509391	0,030315806
P3	PDE7A	-0,412091017	0,000110809	0,008176833
P3	MICOS10	-0,413494945	0,000609802	0,035204041
P3	RPS27L	-0,41450271	0,000712028	0,039898406
P3	CSTB	-0,41493094	0,000167454	0,011718882
P3	TES	-0,415625751	0,000470301	0,02821886
P3	CD53	-0,415926397	3,32389E-08	5,09029E-06
P3	IL18RAP	-0,416382372	0,000797465	0,043499307
P3	PRPF8	-0,418241531	2,12167E-05	0,00187121
P3	ARL6IP5	-0,419468641	1,06132E-06	0,000124106
P3	KIF2A	-0,419655859	5,99374E-07	7,41138E-05
P3	ATRX	-0,419971168	5,08745E-06	0,000505994
P3	NDUFA3	-0,420272738	6,62881E-05	0,005097079
P3	CASP8	-0,421349883	0,000192761	0,013208328
P3	HSPD1	-0,423732668	0,000625942	0,036071465
P3	ARHGAP45	-0,424172819	1,80814E-06	0,000196994
P3	DEK	-0,425377995	1,65616E-05	0,001478468
P3	UBXN4	-0,425536752	1,0931E-05	0,001002721
P3	SEM1	-0,426518619	0,000204443	0,013882774
P3	KPNB1	-0,426590055	5,32393E-05	0,004234675
P3	FGD3	-0,427040368	0,000800689	0,043610142
P3	G3BP1	-0,427630156	6,32828E-05	0,004896857
P3	GDI2	-0,42796874	1,19153E-06	0,000136284
P3	RASSF1	-0,428710639	3,75917E-05	0,003148501
P3	PSIP1	-0,429240078	0,00022133	0,014918795
P3	S1PR4	-0,430866897	0,000491319	0,029383622
P3	CKLF	-0,432276517	9,86346E-07	0,000116081
P3	EIF3A	-0,432911009	1,42092E-05	0,001283638
P3	SCAF11	-0,434075326	1,58277E-05	0,001416406
P3	UBL5	-0,43663162	1,34058E-09	2,55556E-07
P3	ITGB7	-0,436862856	4,98004E-05	0,003988498
P3	DDX39B	-0,439369053	6,47428E-06	0,000625238
P3	FERMT3	-0,439935386	0,00014421	0,010288973
P3	HSPA8	-0,441141516	1,45177E-21	9,16145E-19
P3	ZC3HAV1	-0,442671031	4,04584E-06	0,000412484
P3	SRP9	-0,444462806	5,76526E-07	7,15302E-05
P3	TPR	-0,445372969	4,34903E-05	0,00353731

P3	LNPEP	-0,445497394	3,07157E-05	0,002639025
P3	SMARCE1	-0,445802063	0,000859137	0,046311159
P3	PIK3CD	-0,447984248	0,000652128	0,037236424
P3	HDAC1	-0,449036658	0,000271393	0,017769707
P3	CHD9	-0,450480789	0,000802679	0,043624853
P3	DPYD	-0,450546056	0,000886404	0,047381888
P3	CORO1A	-0,450715691	7,45461E-20	4,13403E-17
P3	SMARCA5	-0,451220959	0,000427469	0,026076313
P3	ADD3	-0,453552037	9,59589E-06	0,000886917
P3	DNAJC8	-0,459120125	0,000412261	0,025402664
P3	CBX3	-0,459562391	0,000231411	0,015484209
P3	ATP5ME	-0,462964952	0,000167032	0,011711782
P3	NRIP1	-0,463397741	0,000448722	0,027236605
P3	NOLC1	-0,464292109	0,00078901	0,043102329
P3	IMP3	-0,466903597	0,00011413	0,008371315
P3	SH3KBP1	-0,467804015	1,29181E-06	0,000146837
P3	TRAV1-2	-0,468613654	2,76568E-11	6,36646E-09
P3	DOCK10	-0,468643665	0,000741002	0,040907108
P3	DGKZ	-0,473904341	7,50275E-07	8,94489E-05
P3	XRCC5	-0,474819601	5,85301E-08	8,60345E-06
P3	TAOK3	-0,478296787	0,000279472	0,018136436
P3	HMGN4	-0,47924605	0,000157357	0,011075806
P3	WDR1	-0,480035007	1,04815E-06	0,000122959
P3	IL7R	-0,481079578	8,24474E-37	7,36014E-34
P3	THEMIS	-0,481993794	3,58778E-05	0,003018765
P3	TMOD3	-0,483719945	0,000155468	0,01096396
P3	PRKDC	-0,4874309	4,37055E-05	0,003546925
P3	DEF6	-0,489285648	8,53727E-06	0,000797124
P3	GYG1	-0,491791666	4,61338E-05	0,003719256
P3	APEX1	-0,493185788	0,00010302	0,007679524
P3	ANXA6	-0,493389517	5,7607E-09	1,00884E-06
P3	TWF2	-0,493702799	0,000319994	0,020382797
P3	PDCD4	-0,497989118	2,62897E-12	6,45792E-10
P3	PRDX1	-0,498351365	3,03056E-05	0,002615502
P3	ZC3H13	-0,498575747	0,000919354	0,048837828
P3	MGAT1	-0,500842988	0,000127665	0,009234497
P3	PRR5	-0,501196206	2,84971E-07	3,73844E-05
P3	SSBP1	-0,502706647	1,42574E-06	0,000159583
P3	METTL23	-0,503693044	0,000430573	0,026221938
P3	SYTL1	-0,518216014	9,32484E-09	1,59485E-06
P3	ARPC5	-0,519749045	3,48525E-08	5,25295E-06
P3	GPR171	-0,520615816	0,000737772	0,040790321
P3	N4BP2L2	-0,520833135	2,9139E-08	4,53836E-06
P3	FBXW5	-0,521562457	7,06924E-05	0,005390446
P3	GBP2	-0,522418797	1,04983E-05	0,000965451
P3	PCSK7	-0,522973299	0,000136291	0,009742972
P3	CLINT1	-0,523022056	5,96743E-06	0,000580888
P3	ZNF638	-0,525032699	0,000712919	0,039898406

P3	SEMA4D	-0,526960611	1,16519E-05	0,001066174
P3	RGS19	-0,528141141	4,66414E-05	0,003751918
P3	LCK	-0,528271139	1,05156E-15	3,49893E-13
P3	ATP6V0E1	-0,529410839	4,10233E-09	7,32435E-07
P3	BAZ1B	-0,530095577	0,000671061	0,037962155
P3	VASP	-0,530265927	0,000358609	0,022398382
P3	CD27	-0,530268431	1,30943E-07	1,80175E-05
P3	RBL2	-0,531719387	1,84844E-12	4,60236E-10
P3	ANTKMT	-0,531959116	0,000278141	0,018114295
P3	SPN	-0,532906711	1,0698E-07	1,50599E-05
P3	ACAP1	-0,533318281	1,42872E-10	3,05805E-08
P3	FNTA	-0,533630729	2,40917E-05	0,002104487
P3	JAML	-0,533649743	8,82642E-06	0,000822025
P3	EIF2S2	-0,534702718	0,000190345	0,01307094
P3	MRPS34	-0,538292229	0,000915783	0,048718868
P3	CHST12	-0,53896004	0,000193067	0,013208328
P3	TUBB	-0,53910929	4,19132E-10	8,52259E-08
P3	CGGBP1	-0,543661416	4,18154E-05	0,003433283
P3	PSMB8-AS1	-0,544296861	0,000197231	0,013417928
P3	GIT2	-0,544416726	0,000931476	0,049410056
P3	CTSC	-0,545440078	5,8033E-09	1,01146E-06
P3	EHD1	-0,545913398	0,000115786	0,008475736
P3	ATP5MC1	-0,549791813	0,000458038	0,027664407
P3	TAP1	-0,550315678	2,37883E-08	3,78554E-06
P3	PPCS	-0,55136466	0,000291142	0,018760699
P3	KLRK1	-0,55157876	1,25927E-08	2,12398E-06
P3	INPP4A	-0,551772296	0,000628251	0,036098302
P3	SP100	-0,552255332	1,83183E-08	2,96668E-06
P3	PDCD10	-0,552549005	0,000732342	0,040655066
P3	ESYT1	-0,552660704	1,25719E-05	0,001144635
P3	KLRG1	-0,55371058	3,12547E-20	1,78743E-17
P3	STK38	-0,555222392	2,33259E-05	0,00204247
P3	SH3BP1	-0,55628562	1,75032E-05	0,001554939
P3	STAT1	-0,55738306	9,57936E-06	0,000886917
P3	LIX1L	-0,557754576	0,00060602	0,035040963
P3	PTK2B	-0,562887371	0,000626798	0,036071465
P3	RCSD1	-0,563352227	5,63954E-09	9,97163E-07
P3	SDAD1	-0,565144777	0,000748745	0,041210267
P3	SAFB	-0,565917015	0,000423882	0,025925875
P3	ANKRD44	-0,566778719	1,51097E-09	2,85218E-07
P3	LYSMD2	-0,573039651	0,000179218	0,012399916
P3	CARD16	-0,57447803	7,66337E-06	0,000721046
P3	PARP10	-0,577041626	0,000593862	0,034501504
P3	LINC00861	-0,57720983	7,14393E-07	8,57296E-05
P3	APOBEC3G	-0,582053781	2,20696E-06	0,000235535
P3	SCML4	-0,583643079	0,000572888	0,033442239
P3	PRPF6	-0,584629953	8,73542E-05	0,00659227
P3	GBP5	-0,585912943	1,91513E-14	5,65288E-12

P3	APBB1IP	-0,5891819	8,2354E-10	1,62932E-07
P3	CASP1	-0,589522064	4,99656E-08	7,43412E-06
P3	UGP2	-0,593787134	0,000186361	0,01284554
P3	LBH	-0,598961055	8,00545E-13	2,06343E-10
P3	YWHAH	-0,605689883	6,60768E-07	8,03481E-05
P3	TNFAIP8	-0,607874393	3,986E-10	8,15038E-08
P3	MDFIC	-0,612190306	1,42389E-05	0,001283638
P3	PCBP1	-0,612411976	1,19899E-14	3,62678E-12
P3	CSK	-0,612844765	1,07387E-10	2,33956E-08
P3	MARCH7	-0,613789737	9,24863E-05	0,006965205
P3	SHFL	-0,614035666	3,16359E-08	4,86515E-06
P3	OIP5-AS1	-0,616038918	0,000457841	0,027664407
P3	EPRS	-0,619612277	3,25353E-05	0,002773866
P3	OGT	-0,622001171	5,15253E-08	7,63513E-06
P3	PDLIM2	-0,622222006	3,03704E-05	0,002615502
P3	POLR2L	-0,626143157	4,27245E-12	1,04251E-09
P3	INO80E	-0,632328153	4,85882E-05	0,003899952
P3	DCK	-0,632355213	0,000515075	0,030593849
P3	MRPL34	-0,632960737	5,61869E-05	0,004413082
P3	CYBC1	-0,633558035	0,000265479	0,01744485
P3	IFI16	-0,636222601	5,6785E-05	0,004433146
P3	NLRC3	-0,636503041	0,00060385	0,034970756
P3	PYHIN1	-0,637331009	7,7254E-07	9,18043E-05
P3	SASH3	-0,638937593	3,84649E-09	6,93524E-07
P3	IRF2	-0,641634941	4,51082E-06	0,000454822
P3	CAP1	-0,645857573	5,0052E-17	1,96984E-14
P3	IRF3	-0,64611119	0,000321759	0,020410254
P3	SESN1	-0,646336496	8,23446E-05	0,006239947
P3	RBX1	-0,647797108	6,47166E-06	0,000625238
P3	KLRD1	-0,649974465	1,81226E-06	0,000196994
P3	TEX264	-0,650096059	0,000239359	0,01595773
P3	TSPAN14	-0,65711534	0,0001886	0,012975466
P3	RGS14	-0,657196045	6,86013E-07	8,28673E-05
P3	LASP1	-0,657811284	0,000272542	0,017813085
P3	ZNF706	-0,658875704	5,47705E-06	0,000538886
P3	TRGC2	-0,660814643	0,000700458	0,039442225
P3	MPHOSPH8	-0,662780583	1,75385E-10	3,68922E-08
P3	LCP2	-0,663824737	2,07124E-21	1,26349E-18
P3	NOP10	-0,66876775	8,37261E-06	0,000785759
P3	IKZF2	-0,669073939	9,06927E-06	0,000842499
P3	PARP1	-0,669457674	1,49978E-07	2,05593E-05
P3	ZBTB38	-0,669973135	1,43128E-06	0,000159714
P3	RPA2	-0,671543896	1,29596E-05	0,001177012
P3	CABIN1	-0,673652947	0,000536179	0,031601746
P3	GRSF1	-0,674984992	1,3413E-05	0,001215172
P3	PSMA5	-0,679710388	1,13114E-07	1,58018E-05
P3	RAB10	-0,681454718	9,29256E-05	0,006969613
P3	SAMHD1	-0,682736099	2,90555E-15	9,16776E-13

P3	CFLAR	-0,682795465	5,55356E-13	1,4519E-10
P3	SARS	-0,685222566	0,000134143	0,009626997
P3	MYC	-0,687400699	3,32639E-05	0,002824808
P3	CCT5	-0,687417984	1,26327E-06	0,00014404
P3	SBNO1	-0,696058214	0,000299736	0,019213034
P3	RASAL3	-0,697681546	2,05033E-11	4,74963E-09
P3	SYNRG	-0,697867811	6,42994E-10	1,29309E-07
P3	GSDMD	-0,698180318	3,51825E-05	0,002967082
P3	ICAM2	-0,705068946	1,26923E-08	2,13097E-06
P3	PLCG1	-0,705750346	6,06191E-08	8,83952E-06
P3	RIPOR2	-0,707042277	2,79886E-19	1,44283E-16
P3	SH3BP5	-0,709208012	2,76857E-05	0,002401246
P3	TBC1D10C	-0,710256875	9,52645E-14	2,66166E-11
P3	LPXN	-0,717354476	9,02113E-11	1,98905E-08
P3	MOB3A	-0,71736604	0,000812696	0,04406738
P3	CCM2	-0,719127715	0,000227594	0,0152847
P3	TIA1	-0,720730424	0,000169887	0,011843859
P3	DOK2	-0,722230136	3,57502E-07	4,64004E-05
P3	SAMD3	-0,725340545	1,82152E-13	5,01275E-11
P3	EGLN2	-0,729089499	0,000323214	0,020431694
P3	LUC7L3	-0,730457187	3,5119E-06	0,000365168
P3	TSC22D4	-0,730739117	4,58781E-11	1,03653E-08
P3	SELPLG	-0,739380181	4,51957E-17	1,81781E-14
P3	HNRNPF	-0,742891431	3,29879E-17	1,35662E-14
P3	FLI1	-0,743288636	1,91811E-13	5,23917E-11
P3	PTGER2	-0,756464839	1,05794E-10	2,31867E-08
P3	BANF1	-0,757070601	1,36743E-08	2,25448E-06
P3	ARL14EP	-0,758853316	8,92582E-07	0,000105385
P3	ACTR3	-0,760809779	1,54249E-16	5,82026E-14
P3	PNP	-0,761930406	1,98612E-08	3,18833E-06
P3	DDX18	-0,766352713	1,32162E-09	2,5326E-07
P3	TRPV2	-0,766930103	3,95545E-05	0,003275418
P3	CD2	-0,773567259	6,21912E-18	2,77593E-15
P3	GLRX	-0,775862455	3,61143E-11	8,26136E-09
P3	PTPN6	-0,781516373	7,89765E-09	1,3635E-06
P3	DDX17	-0,783370256	3,61485E-17	1,47008E-14
P3	MRFAP1L1	-0,783668041	2,37166E-07	3,1638E-05
P3	PPP1R18	-0,791596889	3,00041E-15	9,38616E-13
P3	DCAF7	-0,812409878	2,85184E-06	0,000300807
P3	CYTH4	-0,815133572	0,000180078	0,012435949
P3	FKBP5	-0,81978035	3,95447E-05	0,003275418
P3	RESF1	-0,819853842	1,6046E-24	1,12942E-21
P3	FGFR1OP2	-0,823695898	1,68755E-10	3,57029E-08
P3	HMOX2	-0,8259027	1,97236E-08	3,18019E-06
P3	S100A11	-0,837218463	4,56942E-17	1,81788E-14
P3	IPCEF1	-0,844571054	3,14719E-06	0,000329115
P3	ARHGAP30	-0,8453632	1,1301E-17	4,86621E-15
P3	S1PR1	-0,84683007	2,86252E-10	5,91928E-08

P3	KRI1	-0,851983845	6,85475E-05	0,005248761
P3	DYRK2	-0,853439867	5,68057E-05	0,004433146
P3	AC243960.1	-0,857771218	4,729E-06	0,000474209
P3	CXXC5	-0,869233072	2,89678E-08	4,53098E-06
P3	GBP1	-0,875812709	0,000462142	0,027820468
P3	RASSF7	-0,876072705	0,000642545	0,036804075
P3	ZNF146	-0,879603684	0,000487078	0,029177657
P3	INPP4B	-0,88423425	5,63376E-05	0,004415441
P3	DAXX	-0,889519393	5,11597E-06	0,000507452
P3	MAT2B	-0,89600414	1,77133E-15	5,63762E-13
P3	PPWD1	-0,899515212	0,000257924	0,017009504
P3	CFAP298	-0,901069999	6,5248E-05	0,005027665
P3	CDK2AP2	-0,903718114	1,10833E-09	2,15776E-07
P3	COA3	-0,910257936	0,000660735	0,037493879
P3	TRAF5	-0,912833691	1,8138E-06	0,000196994
P3	ARHGAP25	-0,929351628	4,94283E-10	9,99517E-08
P3	SNX14	-0,930563092	0,000709803	0,039845868
P3	IKZF3	-0,936617076	6,40682E-17	2,49464E-14
P3	ABI3	-0,936807752	2,09963E-07	2,81497E-05
P3	SLAMF6	-0,939380169	5,61732E-05	0,004413082
P3	TRAF3IP3	-0,950008571	1,47578E-25	1,05912E-22
P3	MRPS6	-0,951844454	1,08011E-06	0,000125105
P3	ILK	-0,962915003	7,39985E-06	0,00069985
P3	EOMES	-0,977304578	1,1714E-11	2,74837E-09
P3	PTGDR	-0,984737873	1,66832E-06	0,00018337
P3	PLEK	-0,987947762	2,37508E-18	1,08663E-15
P3	AHSA1	-0,988206744	5,51226E-05	0,004348154
P3	STIP1	-0,988361418	5,71411E-06	0,000560702
P3	NDUFB3	-0,992760956	4,15803E-09	7,38777E-07
P3	CD40LG	-0,99545759	7,0022E-07	8,43051E-05
P3	FOXN2	-0,995758295	4,81094E-08	7,2166E-06
P3	TNFRSF1A	-1,000211	1,24828E-07	1,72409E-05
P3	XPO1	-1,006130815	8,09684E-07	9,59069E-05
P3	RORC	-1,016061902	1,74786E-07	2,36938E-05
P3	VPS35	-1,018199801	0,000127323	0,009228051
P3	NARS	-1,032715201	0,000460852	0,027788559
P3	S100PBP	-1,063001037	0,000849989	0,045953393
P3	SPOP	-1,063936949	0,000636297	0,03650328
P3	DENND2D	-1,068560004	3,79617E-19	1,92217E-16
P3	IFNG-AS1	-1,069479704	0,000830506	0,044966478
P3	ARHGEF2	-1,072956085	0,000320699	0,020382797
P3	LRIF1	-1,074422479	0,00085596	0,046207962
P3	PIM1	-1,075680614	3,72051E-21	2,23237E-18
P3	SIT1	-1,07745254	1,99145E-09	3,69996E-07
P3	TMEM250	-1,099918008	0,000416883	0,025644231
P3	SLFN5	-1,131271005	4,92756E-21	2,90893E-18
P3	ARHGEF3	-1,132890224	2,05764E-12	5,08863E-10
P3	SLC9A3R1	-1,13414979	2,46919E-45	2,58214E-42

P3	CDC42EP3	-1,175209403	6,89509E-23	4,67346E-20
P3	TXNIP	-1,185833812	6,97188E-96	3,18972E-92
P3	ST6GAL1	-1,230815172	2,39415E-08	3,79343E-06
P3	CCR5	-1,267567158	0,000278945	0,018134394
P3	CCR2	-1,325113773	3,96724E-05	0,003277762
P3	MRPS31	-1,341035247	6,7352E-05	0,005168028
P3	GIMAP1	-1,372473598	2,48676E-28	2,02262E-25
P3	GIMAP5	-1,439736128	5,97597E-28	4,75492E-25
P3	GIMAP6	-1,527159095	1,54513E-09	2,90017E-07
P3	GIMAP4	-1,569652557	7,54002E-50	9,85616E-47
P3	SH2D3C	-1,634938955	5,33369E-05	0,004234675
P3	CHMP7	-1,662533998	0,000653498	0,037256527
P3	GIMAP7	-1,673214674	7,19915E-61	1,25474E-57
P3	DBP	-1,765875697	1,7357E-05	0,001545702
P3	CISH	-1,960129738	0,000226165	0,015216669
P4	IER5	0,674489021	4,3402E-09	2,99727E-06
P4	MYADM	0,59997654	3,07104E-07	0,000162903
P4	ITK	0,590217888	1,68971E-06	0,000736249
P4	TUBA4A	0,492081404	0,000195526	0,045582448
P4	AOAH	0,484185249	3,93162E-05	0,01187536
P4	PLCG1	0,475816816	9,31085E-07	0,00044258
P4	GIMAP7	0,470321298	9,1792E-14	1,59985E-10
P4	KCNA3	0,467429161	4,64501E-05	0,013386759
P4	MYH9	0,437228858	1,20119E-18	3,66374E-15
P4	KLF6	0,432612181	2,49103E-12	3,03914E-09
P4	DENND2D	0,431200117	9,86087E-08	5,55258E-05
P4	WNK1	0,43004182	0,000185397	0,044063042
P4	AHNAK	0,424064755	3,62203E-17	9,46928E-14
P4	PPP1R15A	0,405237526	5,86742E-10	4,7723E-07
P4	CCDC88C	0,394964695	2,88334E-06	0,001213025
P4	TUBB	0,392327547	1,86933E-09	1,45573E-06
P4	PRPF8	0,392065614	4,11523E-07	0,000215173
P4	TXNIP	0,381482959	3,41819E-23	1,78727E-19
P4	ATM	0,37627086	3,57483E-08	2,14496E-05
P4	SLFN5	0,375642151	4,184E-06	0,001701539
P4	PIM1	0,371752679	8,18397E-07	0,000404786
P4	MT-ND6	0,351517856	2,24364E-13	3,42164E-10
P4	VPS13C	0,346750826	3,17748E-06	0,001321578
P4	S1PR1	0,343498766	0,000179533	0,042948292
P4	NFKBIA	0,334734559	4,92658E-09	3,33922E-06
P4	LINC00861	0,334380537	0,000138323	0,033978192
P4	SPTAN1	0,332720667	7,95643E-06	0,003033471
P4	GLS	0,324210674	8,97807E-05	0,02347189
P4	MCL1	0,32403934	2,11881E-07	0,000117072
P4	STK10	0,322758943	2,46041E-06	0,001047135
P4	IKZF3	0,321627468	2,39953E-05	0,007772133
P4	AKAP13	0,291589469	8,63049E-05	0,022725503
P4	AKNA	0,265477419	1,43705E-05	0,005106541

P4	IER2	0,264146894	4,19948E-05	0,012296402
P4	MT-ND5	0,252986133	4,80801E-17	1,17319E-13
P4	JUNB	-0,253836751	5,50007E-05	0,015485238
P4	RPL27A	-0,264597356	1,58253E-08	1,01618E-05
P4	LTB	-0,269059539	4,52272E-05	0,013137791
P4	HSPA5	-0,277108699	0,000193405	0,045582448
P4	CIRBP	-0,284606755	1,21833E-06	0,000557402
P4	LSP1	-0,285403669	1,82545E-05	0,006244236
P4	RPL41	-0,28720063	2,13277E-20	9,75769E-17
P4	RPS20	-0,296272337	4,66095E-08	2,70787E-05
P4	EML4	-0,331434995	3,21556E-05	0,00980774
P4	ALDOA	-0,335463524	2,07702E-05	0,006910997
P4	H3F3B	-0,335700452	4,9008E-20	1,99304E-16
P4	LYAR	-0,353534073	0,00010014	0,025631058
P4	SRSF3	-0,358050078	2,10555E-05	0,006942826
P4	FOS	-0,360215753	1,30664E-05	0,004735085
P4	PCBP1	-0,375275761	3,21521E-05	0,00980774
P4	EID1	-0,382482499	0,000135942	0,033618934
P4	LEPROTL1	-0,383757919	2,69933E-05	0,008517089
P4	SNU13	-0,390412569	0,000178884	0,042948292
P4	UCP2	-0,391857803	2,22923E-05	0,007284991
P4	SELENOK	-0,394757092	0,000116183	0,029327029
P4	SAP18	-0,423468113	5,11192E-06	0,002056061
P4	NR4A2	-0,429273158	0,000203721	0,047192257
P4	DDIT4	-0,430418789	1,3391E-05	0,004805154
P4	FTH1	-0,43577373	7,19681E-30	8,78035E-26
P4	STK17B	-0,456876665	3,50242E-09	2,58472E-06
P4	ZFP36L2	-0,458262503	5,29155E-29	4,8419E-25
P4	ZFP36	-0,459940732	2,22376E-11	2,26089E-08
P4	DNAJB1	-0,460605145	5,5893E-10	4,64941E-07
P4	CSRNP1	-0,487249821	1,92251E-05	0,006515359
P4	HLA-C	-0,494400233	2,75079E-53	1,00682E-48
P4	RNPEPL1	-0,495505631	9,23991E-05	0,023816201
P4	HERPUD2	-0,501898766	1,559E-05	0,005434375
P4	MT2A	-0,524433315	4,15442E-05	0,012262564
P4	RNF126	-0,552357078	1,12407E-05	0,004198181
P4	PIK3R1	-0,581618428	9,528E-11	8,71836E-08
P4	CXCR4	-0,583883703	5,83747E-19	1,94234E-15
P4	ITGB1BP1	-0,585781455	0,00015305	0,037345249
P4	CTBP1	-0,612380803	7,42188E-05	0,019974144
P4	TENT5C	-0,6193524	2,968E-08	1,81053E-05
P4	ID2	-0,635984719	2,70146E-14	5,49312E-11
P4	TIPARP	-0,662475586	8,02279E-05	0,021278411
P4	CD81	-0,683498025	9,93714E-14	1,65322E-10
P4	RPS4Y1	-0,71199137	7,35306E-10	5,85063E-07
P4	RBM38	-0,731451333	2,89444E-08	1,79559E-05
P4	PDE4D	-0,748839259	3,91587E-09	2,81029E-06
P4	MAP3K8	-0,85199976	3,65775E-08	2,15931E-05

P4	UBALD2	-0,982635081	3,40831E-13	4,9899E-10
P4	RPS10	-0,986055374	4,95302E-14	9,06427E-11
P4	DUSP2	-1,025806546	1,05189E-48	1,92501E-44
